# FUNDC1 alleviates doxorubicin-induced cardiotoxicity by restoring mitochondrial-endoplasmic reticulum contacts and blocked autophagic flux

**DOI:** 10.7150/thno.92771

**Published:** 2024-06-17

**Authors:** Weibin He, Zhongchan Sun, Guang Tong, Lin Zeng, Wenlong He, Xiaopan Chen, Cien Zhen, Pengyuan Chen, Ning Tan, Pengcheng He

**Affiliations:** 1 Department of Cardiology, Guangdong Provincial People's Hospital (Guangdong Academy of Medical Sciences) Southern Medical University, 510080 Guangzhou, China; 2 Department of Cardiology, Guangdong Provincial Key Laboratory of Coronary Heart Disease Prevention, Guangdong Provincial People's Hospital (Guangdong Academy of Medical Sciences), Guangdong Cardiovascular Institute, 510080 Guangzhou, China; 3 Department of Cardiac Surgery, Guangdong Provincial People's Hospital (Guangdong Academy of Medical Sciences) Southern Medical university, 510080 Guangzhou, China; 4 School of Medicine, South China University of Technology, 510006 Guangzhou, China; 5 Department of Cardiology, Heyuan People's Hospital, 517000 Heyuan, China

**Keywords:** doxorubicin, cardiotoxicity, autophagy, Mitochondrial-Endoplasmic Reticulum Contacts, FUNDC1

## Abstract

**Rationale:** Autophagy dysregulation is known to be a mechanism of doxorubicin (DOX)-induced cardiotoxicity (DIC). Mitochondrial-Endoplasmic Reticulum Contacts (MERCs) are where autophagy initiates and autophagosomes form. However, the role of MERCs in autophagy dysregulation in DIC remains elusive. FUNDC1 is a tethering protein of MERCs. We aim to investigate the effect of DOX on MERCs in cardiomyocytes and explore whether it is involved in the dysregulated autophagy in DIC.

**Methods:** We employed confocal microscopy and transmission electron microscopy to assess MERCs structure. Autophagic flux was analyzed using the mCherry‐EGFP‐LC3B fluorescence assay and western blotting for LC3BII. Mitophagy was studied through the mCherry‐EGFP‐FIS1 fluorescence assay and colocalization analysis between LC3B and mitochondria. A total dose of 18 mg/kg of doxorubicin was administrated in mice to construct a DIC model *in vivo*. Additionally, we used adeno-associated virus (AAV) to cardiac-specifically overexpress FUNDC1. Cardiac function and remodeling were evaluated by echocardiography and Masson's trichrome staining, respectively.

**Results:** DOX blocked autophagic flux by inhibiting autophagosome biogenesis, which could be attributed to the downregulation of FUNDC1 and disruption of MERCs structures. FUNDC1 overexpression restored the blocked autophagosome biogenesis by maintaining MERCs structure and facilitating ATG5-ATG12/ATG16L1 complex formation without altering mitophagy. Furthermore, FUNDC1 alleviated DOX-induced oxidative stress and cardiomyocytes deaths in an autophagy-dependent manner. Notably, cardiac-specific overexpression of FUNDC1 protected DOX-treated mice against adverse cardiac remodeling and improved cardiac function.

**Conclusions**: In summary, our study identified that FUNDC1-meditated MERCs exerted a cardioprotective effect against DIC by restoring the blocked autophagosome biogenesis. Importantly, this research reveals a novel role of FUNDC1 in enhancing macroautophagy via restoring MERCs structure and autophagosome biogenesis in the DIC model, beyond its previously known regulatory role as an mitophagy receptor.

## Introduction

Doxorubicin (DOX)-induced cardiotoxicity (DIC) can severely damage cardiomyocytes and compromise cardiac function, leading to poor prognosis of oncological patients. Up to date, a variety of theories have been postulated for the pathogenesis of DIC, including topoisomerases inhibition, oxidative stress, mitochondrial dysfunction, and dysregulated autophagy 1-3. As the research deepens, the importance of mitochondria has been highlighted and received increasing attention in anthracycline toxicity 4.

Mitochondria and endoplasmic reticulum (ER) form an endomembrane network for their structural and functional communication, which is essential for many cellular functions 5. These physical interactions are termed as MERCs (Mitochondria-Endoplasmic Reticulum Contacts). The past decade has seen dysfunctional MERCs being involved in the pathogenesis of multiple cardiovascular diseases, such as coronary artery disease, heart failure, and pulmonary arterial hypertension 6. Nevertheless, the role of MERCs in the initiation and progression of DIC remained unknown.

FUN14 domain containing 1 (FUNDC1), a mitochondrial outer-membrane protein, was firstly reported as a mitophagy receptor mediating hypoxia-induced mitophagy 7. It was later proposed to be a tethering protein at MERCs fractions, regulating mitochondrial dynamics under hypoxia condition 8. The last few years have witnessed the growing interest in the role of FUNDC1 as a MERCs tethering protein in cardiovascular diseases 9. Interestingly, FUNDC1-meditated MERCs formation was found to be beneficial in dilated cardiomyopathy 10 whereas detrimental in diabetic cardiomyopathy 11. However, whether FUNDC1-meditated MERCs formation was involved in DIC remains unknown.

As a crucial process, macroautophagy (hereafter autophagy) functions as the central mechanism for the degradation of intracellular materials within the lysosome/vacuole and the recycling of macromolecular constituents, which is of great significance for cellular homeostasis 12. Although DOX-induced dysregulated autophagy has been widely studied, a consensus on the role of autophagy in DIC has not yet been reached. Convincing evidence suggests that MERCs exert vital roles in the initiation of autophagy and disruption of MERCs prevents the formation of autophagosomes 13. However, the specific mechanism of dysregulated autophagic flux in DIC and whether MERCs are involved have not been well studied and deserves further investigation.

In the present study, we investigated the detailed status of autophagic flux and explored the possible role of MERCs in DIC. Our results show that autophagic flux was blocked at the early step of autophagy by DOX *in vitro*. Specifically, DOX treatment led to a significant downregulation of FUNDC1 and disruption of MERCs structures, subsequently inhibiting autophagosome biogenesis. Overexpressing FUNDC1 abolished DOX-induced disruption of MERCs and restored the blocked autophagic flux. Mechanistically, FUNDC1 restored autophagosome biogenesis by facilitating ATG5-ATG12/ATG16L1 complex formation, which is essential for LC3 lipidation and autophagosome assembly. Importantly, this restoration depends on FUNDC1's tethering function in maintaining MERCs structures rather than its role as an mitophagy receptor. Furthermore, oxidative stress and cardiomyocyte death in DIC were also alleviated by FUNDC1 overexpression. Notably, cardiac-specific overexpression of FUNDC1 restored the blocked autophagic flux and ameliorated cardiac dysfunction and remodeling induced by DOX *in vivo*. Our results reveal a novel role of FUNDC1 in autophagy by maintaining MERCs structure and facilitating ATG5-ATG12/ATG16L1 complex formation. Furthermore, we demonstrated FUNDC1-meditated autophagy confers protection against DIC.

## Methods

### Reagents and antibodies

Doxorubicin (DOX) (E2516, Selleck Chemicals, Shanghai, China); H2DCF-DA (HY-D0940, MedChemExpress, Shanghai, China); Chloroquine (CQ) (S6999, Selleck Chemicals, Shanghai, China); Bafilomycin A1 (Baf-A1) (S1413, Selleck Chemicals, Shanghai, China.); Histamine (S3968 Selleck Chemicals, Shanghai, China); Primary antibody used in this study: LC3B (18725-1-AP, ProteinTech, Wuhan, China); GAPDH (60004-1-Ig, ProteinTech, Wuhan, China); MFN1 (13798-1-AP, ProteinTech, Wuhan, China); MFN2 (12186-1-AP, ProteinTech, Wuhan, China); IP3R1(8568S, Cell Signaling Technology, Shanghai, China); FUNDC1 (A16318, ABclonal, Wuhan, China); FUNDC1 (A22656, ABclonal, Wuhan, China); FUNDC1 (49240S, Cell Signaling Technology, Shanghai, China); HA (81290-1-RR, ProteinTech, Wuhan, China); HA (66006-2-Ig, ProteinTech, Wuhan, China); TOM20 (11802-1-AP, ProteinTech, Wuhan, China); HSP60 (15282-1-AP, ProteinTech, Wuhan, China); COX IV (11242-1-AP, ProteinTech, Wuhan, China); ATG5 (10181-2-AP, ProteinTech, Wuhan, China); ATG16L1 (67943-1-Ig, ProteinTech, Wuhan, China); DYKDDDDK tag Monoclonal antibody (66008-4-Ig, ProteinTech, Wuhan, China).

### Cell culture and treatment

Human ventricular cardiomyocyte cell lines (AC16) (Cellcook, Guangzhou, China) were grown in DMEM/F12 with 10% fetal bovine serum (FBS). HEK 293T were cultured in DMEM supplemented with 10% FBS. For DOX treatment, cells were treated with 2 μM DOX for 24 h as previously described 14. Special conditions were indicated individually. For CQ treatment, cells were treated 10 μM CQ 2 h prior to further experiments.

### Plasmid construction and stable cell line generation

FUNDC1 sequences were acquired from cDNA dataset using the following primers Forward primer: taccggactcagatctcgagatggcgacccggaaccc; Reverse primer: accgtcgactgcagaattctcagatgcaagtccgagcaaaaag. The FUNDC1 sequences were subsequently inserted into the EcoRI and XhoI sites of Plvx-3×Flag vector (Miaoling, Wuhan, Hubei, China) by using ClonExpress II One Step Cloning Kit (C112, Vazyme biotech, Nanjing, China). The plasmid was sequenced to ensure the plasmid had successfully been constructed. The FUNDC1 truncation was accomplished by Qingke Biotechnology (Beijing, China). Amino acids 7-48 were truncated from full length FUNDC1 to generated mutant FUNDC1 which is unable to promote MERCs formation as previously described 11. The vector plasmid, Plvx-FUNDC1-3×Flag and truncated Plvx-FUNDC1△7-48 were used for lentivirus packaging to generate stably expressed cell line with puromycin selection. For ATG5-HA vector construction, ATG5 sequence was amplified from EGFP-ATG5 (Miaoling, Wuhan. Hubei, China) using the following primers: Forward primer: tctagagctagcgaattcatgacagatgacaaagatgtgc; Reverse primer: acgtcgtatgggtaggatccatctgttggctgtgggat; The ATG5 sequence was further cloned into the vector pLV3-CMV-MCS-3×HA (Miaoling, Wuhan. Hubei, China) using the restricting enzymes XbaI & BamHI and ClonExpress II One Step Cloning Kit (C112, Vazyme biotech, Nanjing, China). pLV3-CMV-ATG5-3×HA was further used for lentivirus packaging and stable cell line generation.

### Endoplasmic Reticulum (ER) and mitochondria contact analysis

Cells were transfected with ER-DsRed plasmid to indicate endoplasmic reticulum while mitochondria were labelled with immunostaining of TOM20. The images were acquired by a confocal microscope (LSM900, Carl Zeiss, Germany). The colocalization of ER with mitochondria was assessed with (Pearson's coefficient, a well-defined and commonly accepted means for describing the extent of overlap between image pairs. Co-localization (Pearson's correlation coefficient) was measured by ZEN software using automatic thresholding.

### Transmission electronic microscopy

The integrity and the morphological change of MERCs were observed by Transmission electronic microscopy (TEM). The samples were fixed by the pre-cooled 2.5% glutaraldehyde and sent to Servicebio Biotechnology Co., Ltd (Wuhan) for further processing and slicing. Images were acquired under a transmission electron microscope (HT7800, HITACHI). For the MERCs quantification (distance between ER and mitochondria within 30 nm), we normalized the ER adjacent to mitochondria to total ER perimeter.

### Autophagic flux measurement (by mCherry-EGFP-LC3B)

Cells were transfected with mCherry-EGFP-LC3B (Miaoling, Wuhan, Hubei, China) Probe using Lipofectamine3000 (Life Technologies). After transfection for 24 h, the cells were plated in confocal dishes for another 24 h before subjected to indicated treatment. Images were acquired with a confocal microscope (LSM900, Carl Zeiss, Germany). The mCherry-only puncta indicated autolysosomes while the yellow puncta in merged image indicated autophagosome. Autolysosomes and autophagosomes per cell were quantified from at least 10 cells from three independent experiments. This analysis was performed by two independent observers, one unaware of sample identity. The autolysosome-to-autophagosome ratio was calculated to indicate autophagic flux.

### Mitophagic flux measurement (by mCherry-EGFP-FIS1)

Cells were transfected with mCherry-EGFP-FIS1 (Miaoling, Wuhan, Hubei, China) Probe using Lipofectamine3000 (Life Technologies). After transfection for 24 h, the cells were plated in confocal dishes for another 24 h before subjected to indicated treatment. Images were acquired with a confocal microscope (LSM900, Carl Zeiss, Germany), followed by manual counting of red-shifted punctae. The red-shifted punctae was quantified from at least 10 cells from three independent experiments. This analysis was performed by two independent observers, one unaware of the identity of the groups.

### Real-time quantitative polymerase chain reaction

RNA was extracted using RNA Extraction Kit (E.N.Z.A.) according to the manufacturer's protocol. The cDNA was synthesized from 1 μg of RNA with M-MLV(AG11706). RT-PCR was performed using the SYBR-Green PCR master mix. (AG11701); Amplification of the sequence of interest was normalized with a reference endogenous gene glyceraldehyde 3-phosphate dehydrogenase (GAPDH). The primers sequences are listed in Table S1-2.

### Immunoblotting

Proteins were fractionated using 8-12% SDS/PAGE gels and transferred to 0.45 μm PVDF. After blocking, the membranes were probed with indicated primary antibody at 4 °C overnight. After incubating with the secondary body for 45 min at room temperature, bands were detected using enhanced chemiluminescence and quantified by FIJI software.

### Measurement of intracellular and mitochondrial calcium

The fluorescent calcium indicator Fluo-4/AM (#F1221, Thermo Fisher Scientific, Waltham, MA) was used to measure changes in intracellular (cytosolic) free Ca^2+^. Cells were incubated with Fluo-4/AM (5 μM) for 30 min at RT in HBSS followed by a 30 min washout in Tyrode's solution (137 mM NaCl, 2.7 mM KCl, 1 mM MgCl_2_, 1.8 mM CaCl_2_, 0.2 mM Na_2_HPO_4_, 12 mM NaHCO_3_, 11 mM glucose, pH 7.4). For cell resting calcium measurement, random five images were acquired by confocal microscope and mean intensity was measured by FIJI software. For calcium handling capacity measurement, the cells were stimulated with 100 μM ATP and images were acquired simultaneously using LSM 900 confocal microscope for 5 min with readings every 5 s. Ca^2+^ transients calculated as ΔF/F0 using FIJI software. The Mito-R-GECO1 was used to indicate mitochondrial calcium level. Mito-R-GECO1 sequence were acquired from plasmid CMV-mito-R-GECO1 (Addgene #46021) using the following primers: Forward primer: taccggactcagatctcgagatgtctgttctgactcctctgc, Reverse primer: cgtcgactgcagaattctccttcgctgtcatcatttgtacaa; and then cloned into Plvx-3×Flag vector (Miaoling, Wuhan, Hubei, China) using the restriction enzyme EcoRI & XhoI and ClonExpress II One Step Cloning Kit (C112, Vazyme biotech, Nanjing, China). The Plvx-Mito-R-GECO1 was further used for lentivirus packaging and stable cell line generation. The Mito-R-GECO1 cell line after indicated treatment was subjected to time series confocal imaging with an interval of 5 s for 5 min. During series image acquisition, cells were stimulated with 200 μΜ histamine (S3968 Selleck Chemicals, Shanghai, China). The intensity of the Mito-R-GECO1 was quantified by FIJI software in each image.

### ATG5 and FUNDC1 knockdown

The shRNA was used to knockdown (KD) ATG5 or FUNDC1.The shRNA sequences target to ATG5 or FUNDC1were cloned into Plko.1 Plasmid. The plasmid was sequenced to ensure the plasmids had successfully been constructed. The negative control plasmid and shRNA plasmids were further used for lentivirus packaging and stable cell line generation. The effect of knockdown was validated by immunoblotting.

The shRNA sequences used in the study are presented below:

Scrambles: 5'-CCTAAGGTTAAGTCGCCCTCG-3'; ATG5 sh1: 5'-GCTTCGAGATGTGTGGTTTGG-3'; ATG5 sh2: 5'-GCAGTGGCTGAGTGAACATCT-3'; ATG5 sh3: 5'-GCTATATCAGGATGAGATAAC-3'; FUNDC1 sh1: 5'-GCCACAGTTCGGGACCTATGG-3'; FUNDC1 sh2: 5'-GCAGCAACTGCAGTAGGTGGT-3'; FUNDC1 sh3: 5'-GCTAGTCATAGTGGCTATGTG -3'.

### Immunoprecipitation assay

Immunoprecipitation assay was performed with Classic Protein A/G Immunoprecipitation Kit (IK-1004, Biolinkedin, Shanghai, China). Briefly, cells after indicated treatment were collected by trypsinization and lysed with IP Lysis buffer for 15 min on ice. The lyses was centrifuged at 15000 rpm for 15 min at 4°C. The supernatant was collected and subjected to concentration measurement by BCA assay.

1 mg proteins were moved into the 1.5 ml EP centrifuge tube and 4 μg anti-HA antibody was added in each tube, followed by incubation overnight at 4°C. The next day, 25 μl Protein A/G Mix Magnetic Beads were added to each tube and incubated in room temperature for 2 h. After that, beads were washed by IP Lysis buffer for three times followed by 10-min boiling in SDS loading buffer.

### Cell viability and cell death measurement

Cell Counting Kit-8 test kits (Dojindo, Japan) were used to determine the vitality of cardiomyocytes according to the manufacturer's guidelines. Cell death was analyzed by SYTOX Green (S7020, Invitrogen) staining. After treatment, cells were stained with 1 μM SYTOX Green dye in 37 °C for 10 min. After incubation, the cells were collected by trypsinization and prepared for flow cytometry analysis (Beckham, USA).

### SOD activity measurement

Total superoxide dismutase (SOD), CuZnSOD, MnSOD were determined utilizing standard assay kits (S0103, Beyotime Biotechnology, Shanghai, China) following the manufacturer's recommendations. Mn-SOD activity was calculated by subtracting GuZn-SOD from total SOD activity.

### MDA measurement

MDA (malondialdehyde) levels were determined using the thiobarbituric acid (TBA)-reactive substance assay using the MDA assay kit (S0131S, Beyotime Biotechnology, Shanghai, China) according to manufacturer's manual. Briefly, TBA was reacted with MDA in lysates at 100 °C for 15 min, Absorbance at 532 nm was measured using a microplate reader (BioTek microplate reader). MDA level was standardized by protein concentration.

### Reactive Oxygen Species (ROS) measurement

ROS level was determined using DCFH-DA probe. Briefly, cells subjected to allocated treatment were incubated with 100 nM DCFH-DA at 37°C for 20 min. After incubation, the cells were collected by trypsinization and prepared for flow cytometry analysis (Beckham, USA).

### Histological analysis

The heart was fixed in 4% paraformaldehyde, embedded in wax and sectioned at 4 µm. For immunohistochemistry (IHC) staining, DAB was used as chromogen and hematoxylin was used to counterstain. To analyze fibrosis, the collagen volume fraction stained with Masson's trichrome stain was analyzed using ImageJ software.

### Animals and treatments

The animal studies were conducted according to the NIH Guide for the Care and Use of Laboratory Animals and approved by the Animal Ethics Committee of Guangdong Academy of Medical Sciences. Male C57BL/6 J mice aged 8 to 10 weeks were used in the present study. All mice were purchased from Guangdong Medical Laboratory Animal Center and provided free access to water and food. Animals were randomly assigned to the following group: Control (CTRL) group (n=10), Doxorubicin (DOX) group (n=10), DOX+FUNDC1 group (n=10). For cardiac-specific FUNDC1 overexpression *in vivo*, a total of 200 μl adeno-associated virus 9 (AAV9) carrying FUNDC1 under the cTnT promoter (AAV9 -*Fundc1*) was injected via tail vein while the CTRL group was injected with AAV9 empty vector via tail vein. The AAV virus were purchased from Hanheng Biotechnology (Hanheng Biotechnology Co., Ltd., Shanghai, China). The DIC model was established as previously described 15. Briefly, 14 days after AAV injection, mice were administrated with DOX (*Selleck*, S1208) at a dose of 6 mg/kg through the tail vein on days 0, 2, and 4. Body Weights were measured on day 0, 14, 18 and 28 since AAV injection. Echocardiography was performed on 14 days after first dose of DOX injection. Mice were anesthetized by inhalation of 1% isoflurane before the cardiac function measurement. Cardiac contractile function represented by left ventricular ejection fraction (LVEF), left ventricular fraction shortening (LVFS) and cardiac output (CO) were calculated by computer algorithms. To determine autophagic flux *in vivo*, half of each group (n=5) was treated with 1.5 mg/kg BafA1 (*Selleck*, S1413) intraperitoneally 2 h before they were euthanized by decapitation. Food was removed 1 hour before BafA1 injection. After euthanasia, the mice hearts were excised and weighted after rinsed in PBS. To measure the tibial length, the tibias were exposed by removing the skeletal muscle and soft tissue.

### TUNEL assay

Terminal deoxynucleotidyl transferase-mediated deoxyuridine triphosphate nick end labeling (TUNEL) staining was used for examining cell apoptosis. The sacrificed heart tissues were embedded in paraffin and cut into 4 μm thick sections. The samples were deparaffinised with xylene and rehydrated with pure ethanol. A 0.5% Triton-X-100 working solution was used for permeabilization, incubated at room temperature for 20 min, and washed thrice with PBS. The TUNEL reaction was performed according to the instructions of the TUNEL Detection Kit (Servicebio, Wuhan, China). The fluorescence of cardiac sections was imaged by confocal microscope (LSM900 Carl Zeiss, Germany).

### Statistical analysis

Independent experiments were repeated at least three independent times. For two-group comparison, two-tailed student t-test was used. For comparisons of three or more groups, we applied one -way ANOVA followed by the Bonferroni post hoc test when the assumptions (equal variance and normal distribution) were satisfied. Otherwise, we used the nonparametric Kruskal-Wallis test followed by the Dunn post hoc test to correct for multiple comparisons. Data are expressed as mean ± SD. Significance was accepted at p < 0.05. Statistical analysis and figure preparation were performed using SPSS (IBM SPSS 23.0, SPSS Inc) and GraphPad Prism 9.0 statistic software, respectively.

## Results

### DOX blocked autophagosome biogenesis in cardiomyocytes

**Figure 1:**
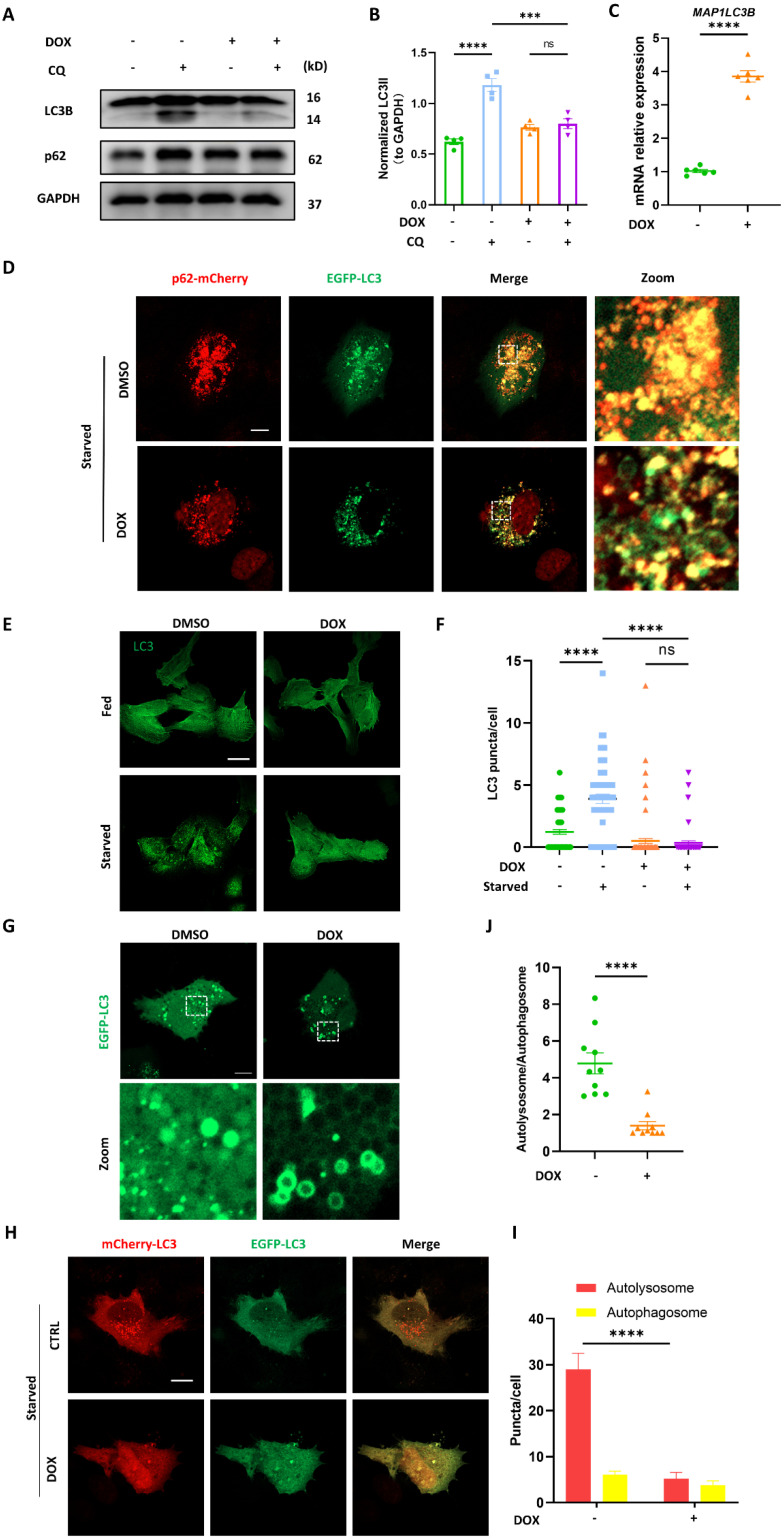
**DOX blocked autophagosome biogenesis in cardiomyocytes. (A)** AC16 were treated with 2 μM DOX for 24 h followed by 2 h CQ treatment, LC3B and p62 were detected with immunoblotting; **(B)** Quantitative analysis of LC3B II (n = 4); **(C)** AC16 were treated with 2 μM DOX for 24 h, mRNA level of MAP1LC3B were detected with qPCR, (n = 6); **(D)** AC16 were cotransfected with p62-mCherry and EGFP-LC3 for 24 h followed by 2 μM DOX treatment for another 24 h and starvation for 2 h. Images were acquired with confocal microscope. Scale bar:5 μm; **(E)** AC16 were treated with 2 μM DOX for 24 h followed by 2 h starvation, LC3 were detected with immunostaining. Scale bar:20 μm **(F)** Quantitative analysis of LC3 puncta (n = 60-90 cells/group); **(G)** AC16 were transfected with EGFP-LC3 for 24 h followed by 2 μM DOX treatment for another 24 h, and starvation 2 h. Images were acquired with confocal microscope. Scale bar:5 μm; **(H)** AC16 were transfected with mCherry-EGFP-LC3B for 24 h followed by 2 μM DOX treatment for another 24 h and starvation for 2 h. Images were acquired with confocal microscope. Scale bar:5 μm; **(I)** Quantitative analysis of numbers of autolysosome and autophagosome (n = 10 cells/group); **(J)** Quantitative analysis the autolysosome-to-autophagosome ratio (n = 10 cells/group). ns: no significance, *** P < 0.001; **** P < 0.0001; DOX: doxorubicin; CQ: chloroquine.

Firstly, chloroquine (CQ), a drug frequently used in autophagy studies as it inhibits the fusion between autophagosomes and lysosomes, was engaged to study autophagic flux. We found that the built-up of LC3B II induced by CQ was significantly reduced by DOX treatment (Figure 1A-B). However, an approximately fourfold increase in *MAP1LC3B* mRNA level was observed in DOX-treated cardiomyocytes (Figure 1C). The discordance between* MAP1LC3B* transcription and protein suggested that DOX might potentially inhibit autophagy by blocking LC3 lipidation (a conversion from LC3 I to LC3 II) rather than *MAP1LC3B* transcription. Besides, CQ induced accumulation of p62 was not observed in DOX-treated cardiomyocytes, suggesting the blocked autophagic flux. Since LC3B lipidation represents an early phase of autophagic flux, we applied starvation to initiate autophagy and evaluated LC3 lipidation by determining its colocalization with p62 and observed that starvation induced substantial colocalization of LC3 and p62, which was inhibited by DOX (Figure 1D). Given the importance of LC3 lipidation in autophagosome synthesis 16, we further investigated autophagosome biogenesis through observing LC3 puncta by immunostaining. Similarly, the increased LC3 puncta induced by starvation were significantly reduced in DOX-treated cardiomyocytes (Figure 1E-F). In addition, we observed a ring-like structure of LC3 puncta in DOX-treated cardiomyocytes transiently expressing EGFP-LC3, suggesting abnormal assembly of autophagosomes (Figure 1G). To systematically evaluate autophagic flux, we used the autophagic flux reporter mCherry-EGFP-LC3 to monitor the dynamics of autophagic flux. The autolysosome-to-autophagosome ratio significantly declined after DOX treatment, and the bigger autophagosomes were observed in DOX-treated cardiomyocytes (Figure 1H-I). These data indicated that autophagic flux was blocked due to either fusion disruption between autophagosome and lysosome or lysosomal dysfunction, consistent with previous studies 17,18. Interestingly, apart from that, we also found that the overall number of LC3 puncta were significantly decreased after DOX treatment, suggesting that autophagosome biogenesis was also impaired (Figure 1J). Taken together, these results indicated that autophagosome biogenesis was blocked in DOX-treated cardiomyocytes.

### DOX interrupted mitochondria-endoplasmic reticulum contacts formation

**Figure 2:**
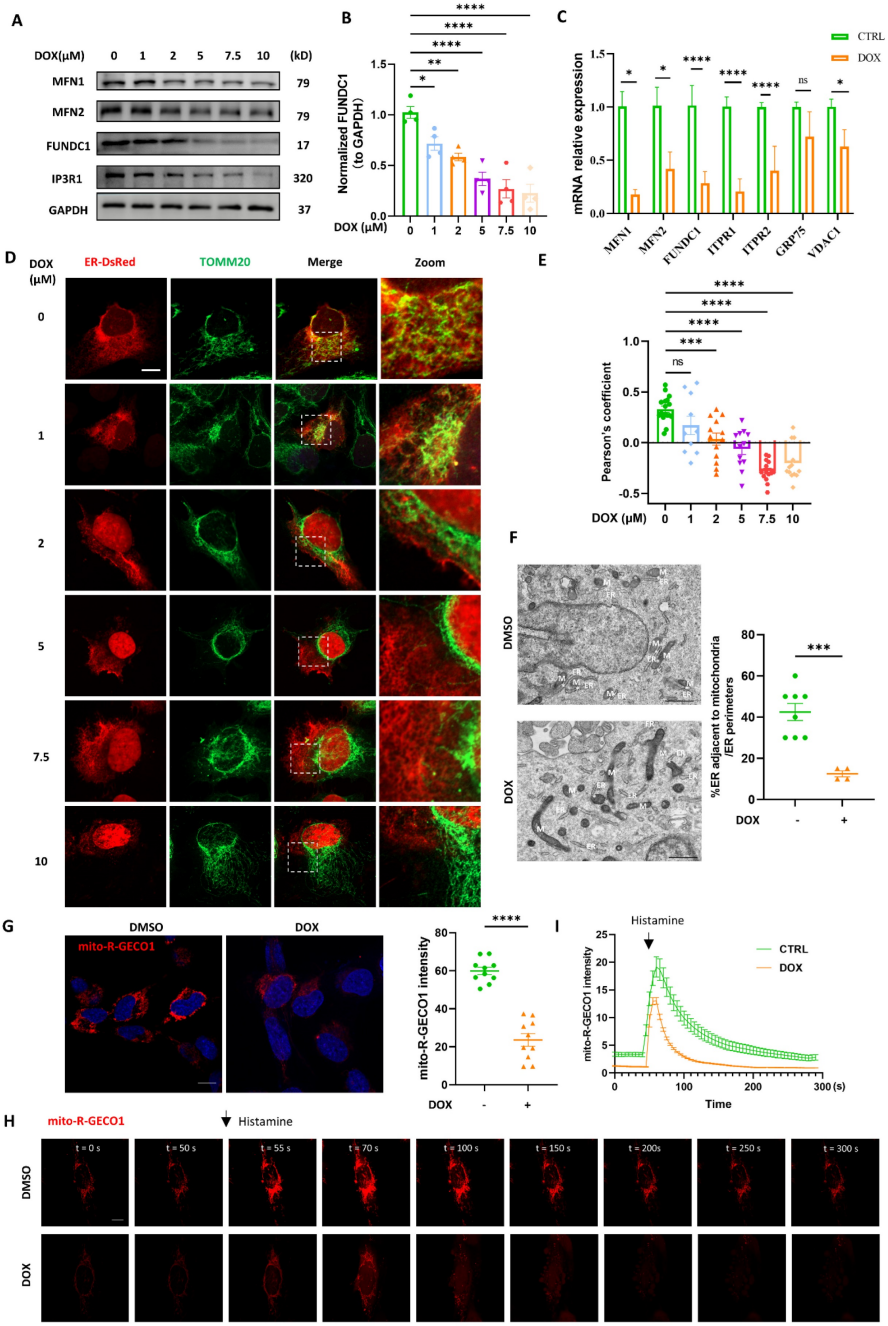
** DOX interrupted mitochondria-endoplasmic reticulum contacts formation. (A)** AC16 were treated with indicated concentration of DOX for 24 h, MFN1, MFN2, FUNDC1 and IP3R1 were detected with immunoblotting; **(B)** Quantitative analysis of expression of FUNDC1 (n = 4); **(C)** AC16 were treated with 2 μM DOX for 24 h, mRNA level of MFN1/2, FUNDC1, IP3R1/2, VDAC1 and GRP75 were detected with qPCR; **(D)** AC16 were transfected with ER-DsRed for 24 h followed by different concentrations of DOX treatment for another 24 h, immunostaining of TOM20 indicated mitochondria, images were acquired with confocal microscope. Scale bar:5 μm; **(E)** Quantitative analysis of Pearson's coefficients indicating colocalization of mitochondria and endoplasmic reticulum (n = 10-17 cells/group); **(F)** Transmission electron microscope analysis of mitochondria and endoplasmic reticulum, M: mitochondria, ER: endoplasmic reticulum, asterisk indicated MERCs. Scale bar:500 nm. MERCs formation was quantified; **(G)** mito-R-GECO1 stable cell line was subjected to 24 h DOX treatment, images were acquired with confocal microscope, Scale bar:20 μm; mito-R-GECO1 intensity was quantified; **(H)** mito-R-GECO1 stable cell line was subjected to 24 h DOX treatment, cells were stimulated with 0.2 mM histamine, followed by time series imaging by confocal microscope. Scale bar:5 μm; **(I)** The intensity of mito-R-GECO1 over time was quantified (n = 6 cells/group); ns: no significance; * P < 0.05; ** P <0.01; *** P < 0.001; **** P < 0.0001, DOX: doxorubicin.

Mitochondria and ER contacts (MERCs) are where autophagy initiates and autophagosomes form 13. We wondered whether autophagosomes biogenesis inhibition induced by DOX was attributed to the disrupted MERCs structure. MERCs mainly contain several tethers: MFN1/MFN2 heterodimer; MFN2 homodimers; VDAC-GRP75-IP3R; and FUNDC1-IP3R 19. We found that multiple MERCs tethering proteins, including MFN1, MFN2, FUNDC1, and IP3R were significantly downregulated by DOX in a dose-dependent manner. Among these MERCs proteins, FUNDC1 stood out most prominently (Figure 2A). Quantification analysis indicated that FUNDC1 was significantly decreased by DOX treatment at a minimum concentration of 1 μM, while other tethering proteins were unaffected at this concentration (Figure 2B, Figure S1A-C). Therefore, FUNDC1 was chosen for the following study. DOX also downregulated MFN1/2, FUNDC1, IP3R1 expression in a time-dependent manner (Figure S1D). Besides, the transcriptional level of MERCs components was also determined. We also observed a significant reduction in the transcriptional level of MFN1/2, FUNDC1, VDAC1, and IP3Rs upon 2 μM DOX treatment (Figure 2C). Given the significant downregulation of MERCs' tethers, we further evaluated MERCs formation by confocal imaging through labeling mitochondria and endoplasmic reticulum (ER), respectively. We found that the MERCs structures were disrupted by DOX at a dose-dependent manner, as demonstrated by the decreased colocalization of mitochondria and ER (Figure 2D-E). The disruption of MERCs was further validated by transmission electron microscopy (TEM). DOX treatment led to an increased spatial distance between mitochondria and ER, indicating the inhibitory effect of DOX on MERCs formation (Figure 2F). Considering the important role of MERCs in calcium handling, we also determined the intracellular calcium level. Surprisingly, DOX treatment did not lead to a change in resting intracellular calcium level (Figure S1E-F), whereas it did result in a significant lower calcium peak induced by ATP stimulation and a delayed recovery of the calcium to the baseline level (Figure S1G). These findings suggested compromised calcium handling capacity in DOX-treated cardiomyocytes, which may be potentially associated with disruption of MERCs structure. To further evaluate whether the disrupted MERCs structure induced by DOX blocked the calcium transfer from ER to mitochondria, we generated cardiomyocytes stably expressing mitochondrial-targeted calcium indicator (Mito-R-GECO1) 20. The fluorescence intensity of Mito-R-GECO1 reflects the Ca^2+^ level in mitochondria. We found that mitochondrial Ca^2+^ level was significantly decreased by DOX treatment (Figure 2G), which might be potentially attributed to the disrupted MERCs structure. To validate this, we evaluated mitochondrial calcium dynamics as we previously described 21. Rapid mitochondrial Ca^2+^ influx was induced by histamine treatment. We found that the amplitude of mitochondrial Ca^2+^ transients induced by histamine were significantly compromised in DOX-treated cardiomyocytes (Figure 2H-I). Overall, these data demonstrated that DOX disrupted MERCs structure and compromised its function by damaging ER and mitochondria tethering.

### FUNDC1 overexpression restored disrupted MERCs structure in DOX-treated cardiomyocytes

**Figure 3:**
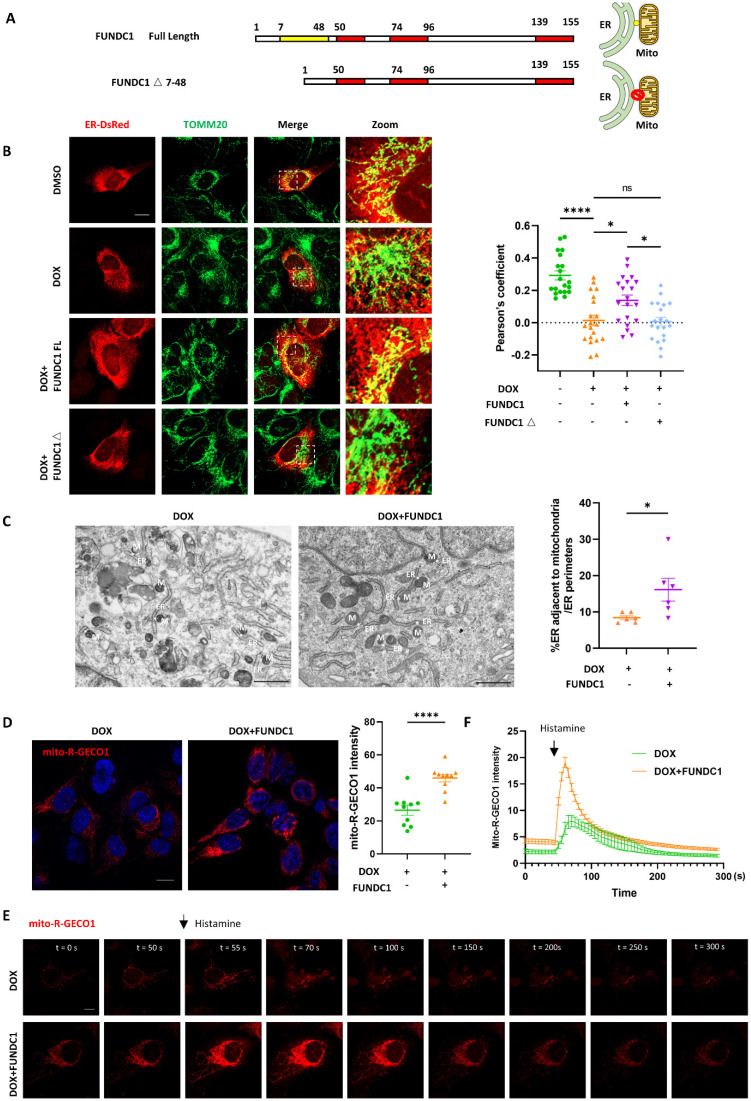
** FUNDC1 overexpression restored MERCs structures in DOX-treated cardiomyocytes. (A)** Scheme graph indicated that full length FUNDC1 domain in which red domain represent transmembrane domain and yellow domain represent the AA7-48 necessary for tethering mitochondria to ER. The FUNDC1△ 7-48 indicated truncated FUNDC1 which lack AA 7-48 and is supposed to be unable to promote MERCs formation; **(B)** Cardiomyocytes were transfected with ER-DsRed for 24 h followed by 2 μM DOX treatment for 24 h, immunostaining of TOM20 indicated mitochondria, images were acquired with confocal microscope, Scale bar:5 μm, Pearson's coefficients indicating colocalization of mitochondria and endoplasmic reticulum were quantified, (n = 10-17 cells/group); **(C)** Transmission electron microscope analysis of mitochondria and endoplasmic reticulum, M: mitochondria, ER: endoplasmic reticulum, asterisk indicated MERCs. Scale bar:500 nm. MERCs formation was quantified; **(D)** mito-R-GECO1 stable cell line stably expressing vector or FUNDC1 were subjected to 24 h DOX treatment, images were acquired with confocal microscope. Scale bar:20 μm; mito-R-GECO1 intensity was quantified; **(E)** mito-R-GECO1 stable cell line stably expressing vector or FUNDC1 were subjected to 24 h DOX treatment, cells were stimulated with 0.2 mM histamine, followed by time series imaging by confocal microscope. Scale bar:5 μm; **(F)** The intensity of mito-R-GECO1 over time was quantified (n = 6 cells/group); ns: no significance; * P < 0.05; **** P < 0.0001, DOX: doxorubicin.

Since the downregulation of FUNDC1 was prominent in DOX-treated cardiomyocytes, we investigated whether restoration of FUNDC1 was able to alleviate MERCs disruption. FUNDC1 overexpression itself didn't increase the colocalization of mitochondria and ER, but it mitigated the damage of MERCs structure caused by DOX (Figure S2A-B). To further verify the role of FUNDC1 on MERCs restoration, we constructed a truncated mutant of FUNDC1 lacking N-terminal AA7-48 (termed FUNDC1△), which was unable to tether mitochondria with ER 11 (Figure 3A). Interestingly, the truncated FUNDC1 failed to restore the disrupted MERCs structures induced by DOX treatment (Figure 3B). To exclude the possibility that the truncation of FUNDC1 renders it loss of mitochondrial localization and subsequent failure of tethering mitochondria to ER, we also determined its subcellular localization. We found that FUNDC1△ was mostly colocalized with mitochondria as full length FUNDC1 did (Figure S2C). The effect of FUNDC1 overexpression on MERCs restoration was also confirmed by TEM (Figure 3C). Furthermore, the calcium dynamics was determined as described above. Although FUNDC1 did not alter the resting cytosolic calcium in DOX-treated cardiomyocytes (Figure S2D-E), it significantly enhanced the amplitude of cytosolic calcium transients induced by ATP (Figure S2F). Moreover, FUNDC1 overexpression restored the reduced mitochondrial calcium and the amplitude of mitochondrial calcium transients induced by histamine in DOX-treated cardiomyocytes (Figure 3D-F). The overexpression of FUNDC1 in Mito-R-GECO1 stably expressed cell line was validated by immunoblotting. (Figure S2G). These findings pointed to that overexpression of FUNDC1 corrected DOX-induced calcium disturbance. To summarize, all these data demonstrated that FUNDC1 downregulation primarily contributed to the disrupted MERCs structure and impaired MERCs function in DOX-treated cardiomyocytes, which can be restored by FUNDC1 overexpression.

### Restoration of MERCs structure by FUNDC1 ameliorated the impaired autophagosome biogenesis in DOX-treated cardiomyocytes

**Figure 4:**
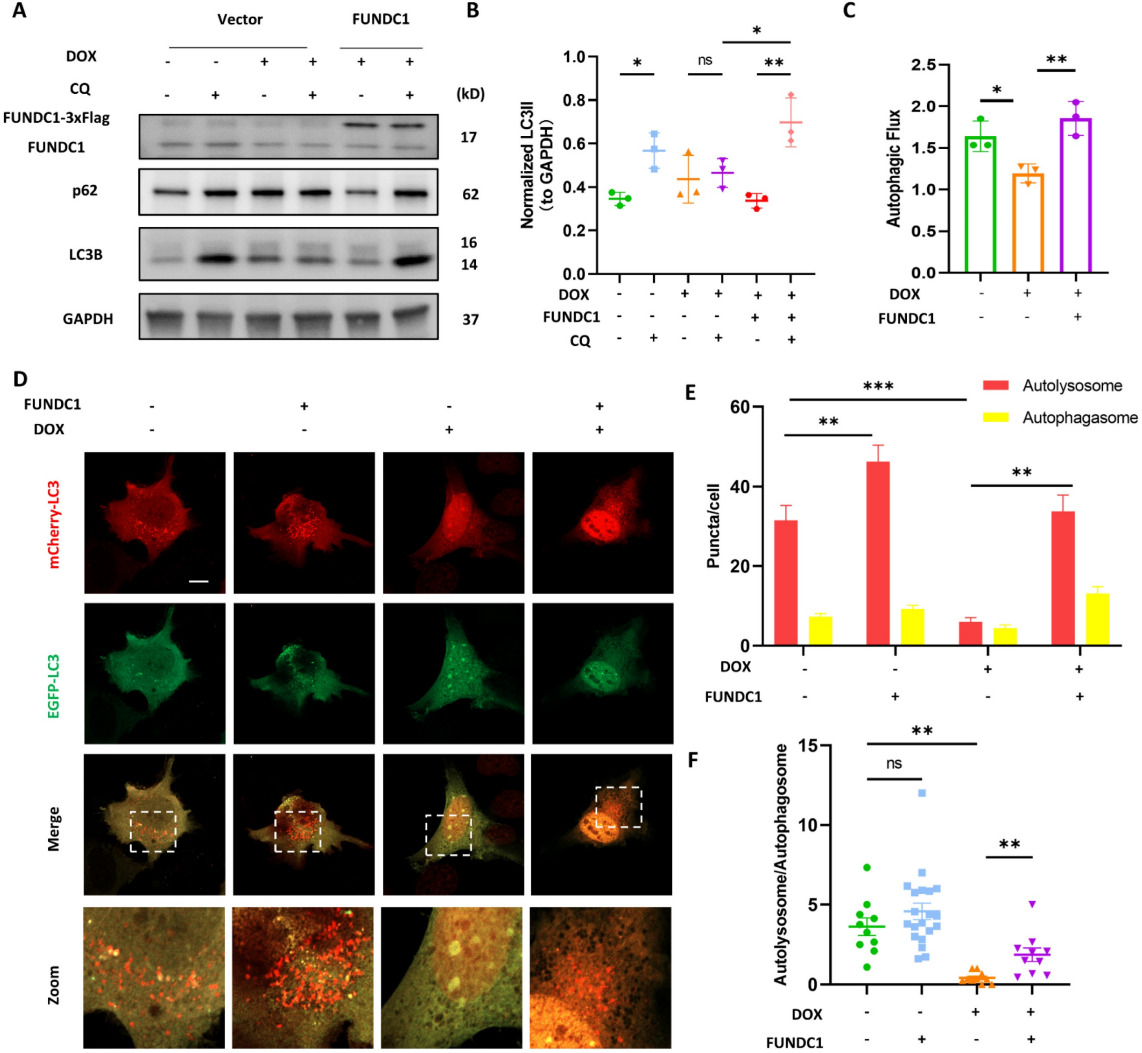
** FUNDC1 overexpression restored the blocked autophagic flux in DOX-treated cardiomyocytes. (A)** AC16 were transfected with Vector or FUNDC1 plasmid for 24 h followed by 2 μM DOX treatment for 24 h and CQ treatment for 2 h. LC3B, p62 and FUNDC1 were detected with immunoblotting; **(B)** Quantitative analysis of LC3B II (n = 3); **(C)** Autophagic flux was calculated from the change in normalized LC3-II levels to GAPDH upon CQ treatment in left panel, (n = 3); **(D)** AC16 were transfected with mCherry-EGFP-LC3B for 24 h followed by 2 μM DOX treatment for 24 h and starvation for 2 h. Images were acquired with confocal microscope. Scale bar:5 μm; **(E)** Quantitative analysis of numbers of autolysosome and autophagosome (n = 10-20 cells/group); **(F)** Quantitative analysis the ratio of autolysosome and autophagosome. (n = 10-20 cells/group); ns: no significance; * P < 0.05; ** P <0.01; *** P < 0.001, DOX: doxorubicin.

Given that MERCs are known as locations where phagophore forms and the damaged MERCs can be corrected by FUNDC1 overexpression, we hypothesized that overexpression of FUNDC1 could restore the blocked autophagic flux induced by DOX. We found that CQ-induced LC3B II accumulation was significantly increased upon FUNDC1 overexpression (Figure 4A-B). Autophagic flux, as indicated by LC3B II turnover, was recovered by FUNDC1 overexpression (Figure 4C). Besides, p62 accumulation in response to CQ treatment reappeared upon FUNDC1 overexpression (Figure 4A), suggesting the restored autophagic flux. We also applied mCherry-EGFP-LC3 reporter to evaluate autophagic flux and observed that FUNDC1 overexpression significantly restored the reduced overall puncta induced by DOX under starvation conditions (Figure 4D-E). Furthermore, the decreased autolysosome-to-autophagosome ratio induced by DOX was also markedly augmented by FUNDC1 overexpression (Figure 4F), suggesting that FUNDC1 overexpression restored the blocked autophagic flux in DOX-treated cardiomyocytes. To explore whether the restoration of autophagic flux by FUNDC1 depended on its effect on MERCs formation, we applied the FUNDC1△ generated above to study the autophagic flux and showed that FUNDC1△ failed to restore the reduced CQ-induced LC3B II accumulation as full length FUNDC1 did (Figure S3A-B). Taken together, these results indicated that FUNDC1 restored the blocked autophagic flux in DOX-treated cardiomyocytes, depending on its function on MERCs formation.

### FUNDC1 enhanced autophagosome biogenesis by facilitating ATG5-ATG12/ATG16L1 complex formation

To further clarify the effect of FUNDC1 on LC3B II was due to the increased LC3 transcription or promotion of LC3 lipidation, we also determined the mRNA level. Surprisingly, the increased transcription of *MAP1LC3B* induced by DOX was mitigated by FUNDC1 (Figure S3C). These results abrogated the possibility that FUNDC1 facilitated LC3B II production by promoting *MAP1LC3B* transcription, and instead indicated that FUNDC1 may restore the autophagic flux by facilitating LC3 lipidation. LC3 lipidation is necessary for autophagosome biogenesis, in which the formation of ATG5-ATG12/ATG16 complex plays a crucial role 22.

**Figure 5:**
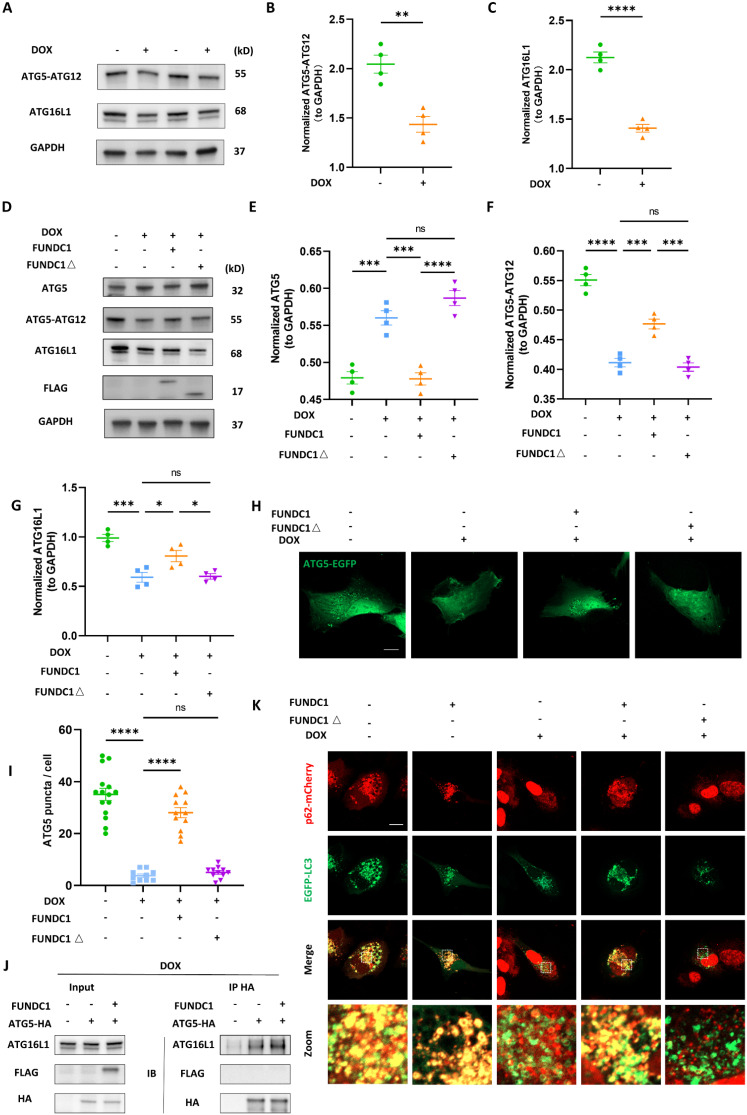
** FUNDC1 enhanced autophagosome biogenesis by facilitating ATG5-ATG12/ATG16L1 complex formation**. **(A)** AC16 were treated with indicated concentration of DOX for 24 h, ATG5-ATG12 and ATG16L1 were detected with immunoblotting; **(B)** Quantitative analysis of ATG5-ATG12 (n = 4); **(C)** Quantitative analysis of ATG16L1 (n = 4); **(D)** FUNDC1 or FUNDC1△ stable cell line were subjected to DOX treatment for 24 h, ATG5, ATG5-ATG12 and ATG16L1 were detected with immunoblotting; **(E)** Quantitative analysis of ATG5 (n = 4); **(F)** Quantitative analysis of ATG5-ATG12 (n = 4); **(G)** Quantitative analysis of ATG16L1 (n = 4); **(H)** FUNDC1 or FUNDC1△ stable cell line were transfected with EGFP-ATG5 for 24 h followed by 2 μM DOX treatment for 24 h and starvation for 2 h, Images were acquired with confocal microscope. Scale bar:5 μm; **(I)** Quantitative analysis of ATG5 puncta (n = 11-15 cells/group); **(J)** ATG5-HA or/and FUNDC1 stable cell line were subjected to DOX treatment for 24 h. Immunoprecipitation were performed anti-HA antibody followed by immunoblotting of ATG16L1; **(K)** Full length FUNDC1 and FUNDC1△ stable cell line were co-transfected with p62-mCherry and EGFP-LC3 for 24 h followed by 2 μM DOX treatment for 24 h and starvation for another 2 h. Images were acquired with confocal microscope. Scale bar:5 μm; ns: no significance; *** P < 0.001; **** P < 0.0001, DOX: doxorubicin.

To this end, we determined the ATG5-ATG12 complex and ATG16L1 in DOX-treated cardiomyocytes and found that DOX led to the decrease of ATG5-ATG12 complex and ATG16L1 level (Figure 5A-C). Accordingly, DOX treatment also blocked starvation-induced ATG5 puncta formation (Figure S3D). The activity of phosphatidylinositol-3-phosphates (PI3P) complex is prerequisite for recruiting the ATG5-ATG12/ATG16L1 complex 23. Thus, we also applied EGFP- FYVE probe to determine the activity of PI3P complex 24. Under fed conditions, EGFP-FYVE showed a pattern of diffuse distribution. However, upon starvation, the probe formed big puncta, which were inhibited by DOX, indicating impaired PI3P activity (Figure S3E). Given that FUNDC1 was able to enhance LC3 lipidation in DOX-treated cardiomyocytes, we determined the effect of FUNDC1 on ATG5-ATG12/ATG16L1 complex formation. Indeed, FUNDC1 overexpression significantly decreased ATG5 monomer and augmented ATG5-ATG12 and ATG16L1 level (Figure 5D-G), while FUNDC1 knockdown failed to exert similar effects (Figure S4A-D). Interestingly, the restored ATG5-ATG12 and ATG16L1 was dependent of the tethering domain of FUNDC1 since FUNDC1△ failed to achieve the same effect (Figure 5D-G). Accordingly, FUNDC1 but not FUNDC1△ restored ATG5 puncta formation in DOX-treated cardiomyocytes (Figure 5H-I).

To further validate that FUNDC1 facilitated the formation of ATG5-ATG12/ATG16L1 complex and subsequent LC3 lipidation. We labeled the ATG5 with HA on its C-terminal and then pulled down ATG16L1 with HA tag. We found that FUNDC1 overexpression increased the ATG16L1 pulled down by ATG5-ATG12 (Figure 5J), suggesting that FUNDC1 overexpression boosted ATG5-ATG12/ATG16 complex formation. It was worth noting that FUNDC1 itself did not interact with ATG5-ATG12 directly (Figure 5J). To evaluate LC3 lipidation, we expressed p62 and LC3B simultaneously and assessed their colocalization. Our result demonstrated that the blocked p62 and LC3 colocalization by DOX was recovered by FUNDC1 overexpression, whereas FUNDC1△ failed to exert similar effects (Figure 5K). Besides, FUNDC1 also restored the impaired PI3P activity in DOX-treated cardiomyocytes (Figure S3F). Taken together, these results indicated that FUNDC1 restored the autophagosome biogenesis by facilitating the formation of ATG5-ATG12/ATG16L1 complex and subsequent autophagosome assembly process.

### FUNDC1 overexpression did not alter the mitophagic flux in DOX-treated cardiomyocytes

Apart from acting as a MERCs tethering protein, FUNDC1 also serves as a mitophagy receptor, recruiting LC3-positive autophagosome to engulf mitochondria. Therefore, we explored whether FUNDC1 overexpression affected mitophagic flux in DOX-treated cardiomyocytes. Our data showed that there was no significant recruitment of LC3-positive autophagosomes to mitochondria upon DOX treatment (Figure S5A). Although FUNDC1 overexpression itself induced significant recruitment of autophagosomes to mitochondria, it failed to exert similar roles in DOX-treated cardiomyocytes (Figure S5A). Besides, we transduced cardiomyocytes with a sensitive mitophagy sensor based on a tandem mCherry-EGFP-FIS1 construct. This sensor is anchored in the mitochondrial membrane and undergoes a red shift due to lysosomal quenching of the acid-sensitive EGFP moiety once mitochondria are delivered to lysosomes through mitophagy. Microscopic analysis of the number of red-shifted puncta, indicative of mitophagy events, revealed a significant increase in lysosomal delivery of damaged mitochondria in FUNDC1-overexpressed cardiomyocytes (Figure S5B). However, this effect was abolished in cardiomyocytes co-treated with DOX, indicating that the effect of FUNDC1 on mitophagic flux might be context-dependent. (Figure S5B). Furthermore, we detected multiple mitochondrial proteins to evaluate the degradation of mitochondria. Notably, the mitochondria proteins including HSP60, COX IV and TOM20 were significantly decreased upon FUNDC1 overexpression, suggesting increased degradation of mitochondria by mitophagy (Figure S5C-D). These findings validated the role of FUNDC1 as a mitophagy receptor, as reported by previous study 7. However, the degradation of mitochondria by FUNDC1 overexpression was not observed in DOX-treated cardiomyocytes. Taken together, our results indicated that FUNDC1 did not alter the mitophagic flux in DOX-treated cardiomyocytes.

### FUNDC1 overexpression alleviated DOX-induced oxidative stress and cardiomyocytes death in an autophagy-dependent manner

**Figure 6:**
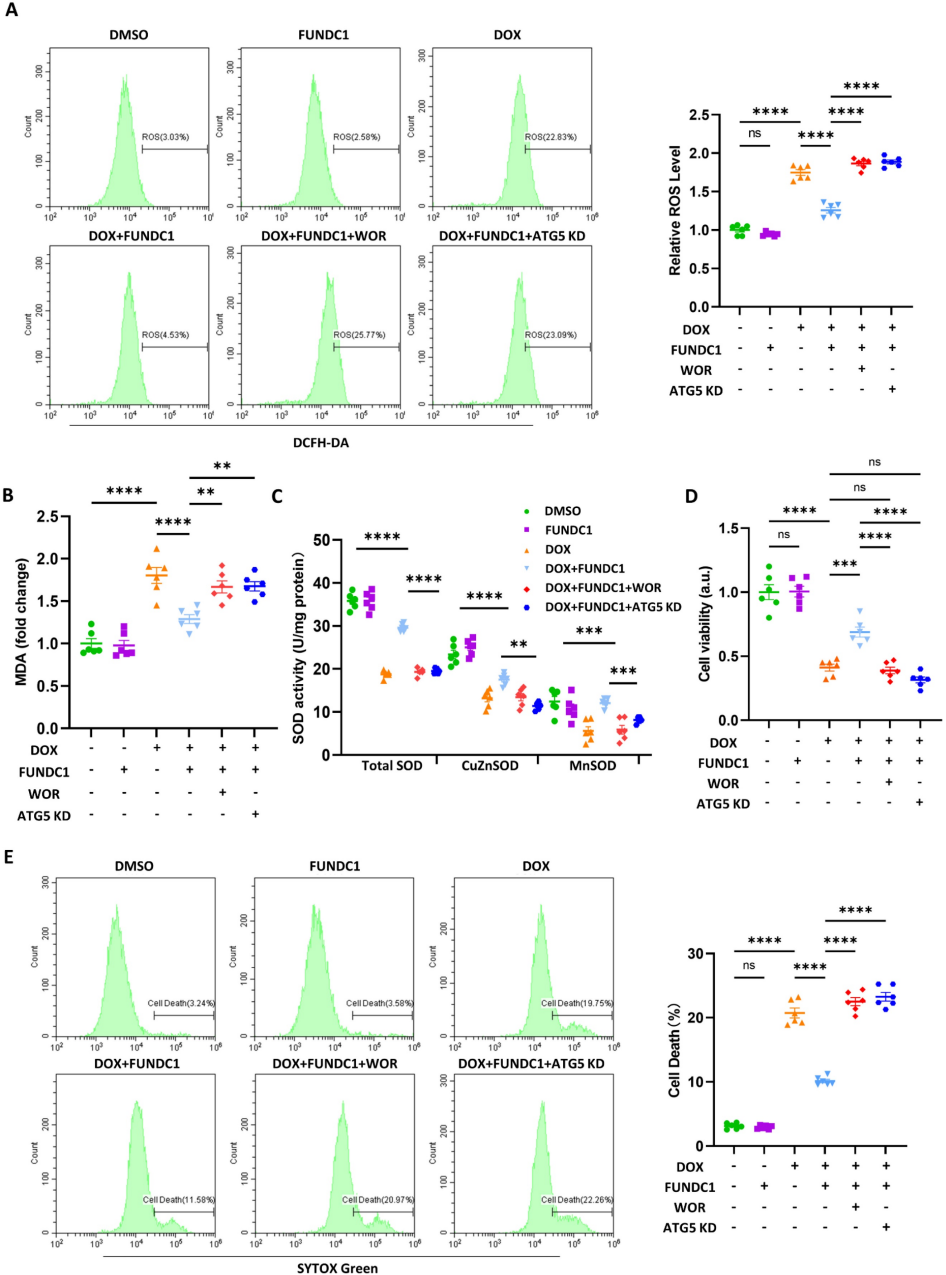
** FUNDC1 overexpression alleviated DOX-induced oxidative stress and cardiomyocytes death in an autophagy-dependent manner.** Negative control or ATG5 knock-down (ATG5 KD) cell lines were treated with 2 μM DOX or/and 10 μM wortmannin for 24 h. **(A)** ROS was detected by DCFH probe, relative ROS level in each group was quantified, (n = 6); **(B)** Relative MDA level in each group, (n = 6); **(C)** SOD activities were determined in each group. (n = 6); **(D)** Cell viability was determined by CCK-8 assay (n = 6); **(E)** Cell death was determined by SYTOX Green staining (n = 6); ns: no significance; ** P <0.01; *** P < 0.001; **** P < 0.0001, DOX: doxorubicin.

To explore the effect of FUNDC1-meditated autophagy on DOX-induced cardiomyocytes injury, we assessed DOX-induced oxidative stress and cardiomyocytes death after overexpressing FUNDC1. Our result indicated that DOX significantly increased the generation of reactive oxygen species (ROS), which was alleviated by FUNDC1 overexpression and aggravated by FUNDC1 knock-down (Figure 6A, Figure S6A-B). To determine whether autophagy was involved we blocked the autophagy either pharmacologically by PI3P inhibitor wortmannin or genetically by ATG5 knock-down.

The efficacy of knock-down was validated by immunoblotting (Figure S6C). We found that both wortmannin administration and ATG5 knock-down abolished the protective effect of FUNDC1 overexpression (Figure 6A). Similarly, the level of malondialdehyde (MDA), another oxidative stress indicator, was also reduced by FUNDC1 overexpression and elevated by FUNDC1 knock-down. (Figure 6B, Figure S6D). Besides, FUNDC1 overexpression also conferred an antioxidant effect against DIC in autophagy-dependent manner, as demonstrated by the activity of cellular and mitochondrial superoxide dismutase (SOD) (Figure 6C). All above effects were reversed by FUNDC1 knock-down (Figure S6E). More importantly, FUNDC1 overexpression ameliorated cell viability reduction and cell deaths induced by DOX, the effects of which were abolished by FUNDC1 knock-down (Figure 6D-E, Figure S6F-H). Administration of wortmannin and knock-down of ATG5 also verified the involvement of autophagy in the protective effects of FUNDC1. Overall, these data indicated that FUNDC1 overexpression alleviated DOX-induced oxidative stress and cardiomyocytes death in an autophagy-dependent manner.

### Cardiac-specific overexpression of FUNDC1 restored disrupted MERCs structures and blocked autophagic flux *in vivo*

**Figure 7:**
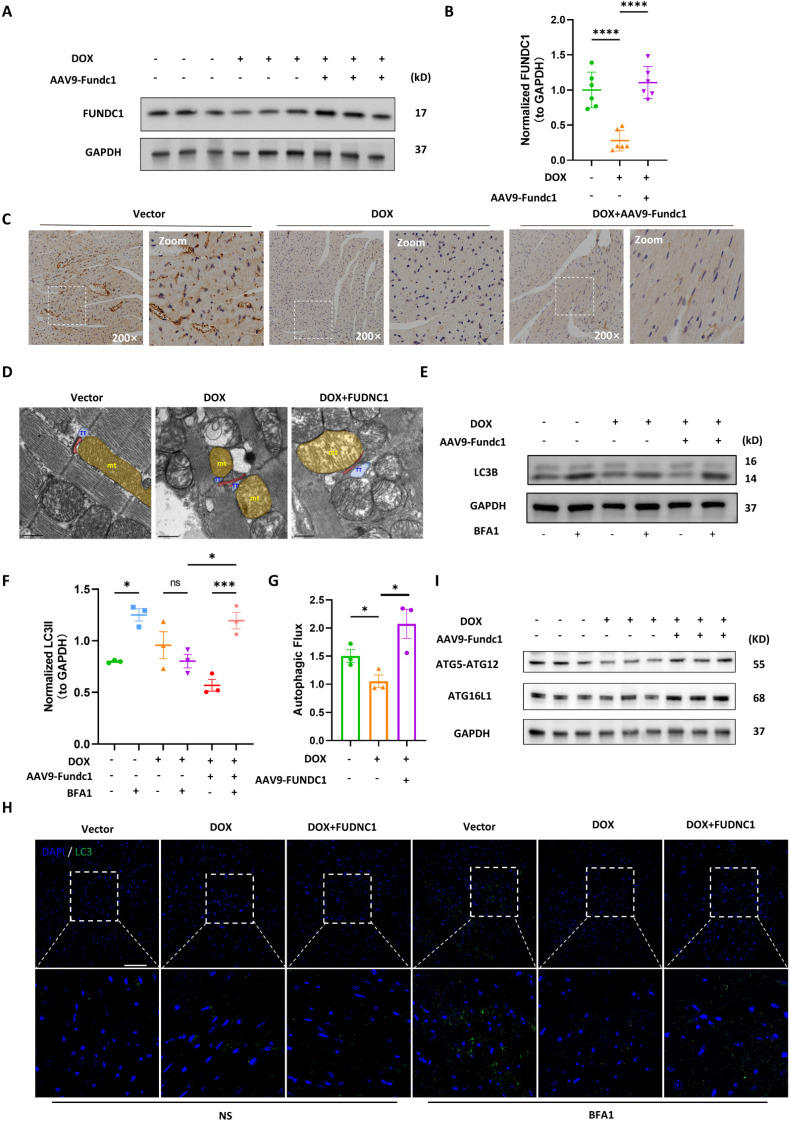
** Cardiac-specific overexpression of FUNDC1 restored MERCs structures and blocked autophagic flux *in vivo*. (A)** Immunoblotting of FUNDC1 in heart tissue; **(B)** Quantitative analysis of FUNDC1 expression in heart tissue, (n = 6); **(C)** Immunohistochemistry staining of FUNDC1 in heart tissue; **(D)** Representative TEM images of MERCs in heart tissue, mt: mitochondria-yellow, TT: transversal tubule-blue; junctional sarcoplasmic reticulum (jSR)-red; **(E)** Immunoblotting of LC3B in heart tissue; **(F)** Quantitative analysis of LC3B II(n = 3); **(G)** Autophagic flux was calculated from the change in normalized LC3-II levels to GAPDH upon BFA1 treatment in left panel, (n = 3). **(H)** Representative images of immunostaining of LC3 in heart tissue. ns: No significance, Scale bar:20 μm; **(I)** Immunoblotting of ATG5-ATG12 and ATG16L1 in heart tissue; ns: no significance; * P < 0.05; *** P < 0.001; **** P < 0.0001, DOX: doxorubicin; NS: Normal saline; BFA1: Bafilomycin A1.

To evaluate the cardioprotective effect of FUNDC1 against DIC *in vivo*, we applied adeno-associated virus (AAV) 9 to overexpress FUNDC1 in myocardium, and a DIC animal model was constructed as previously described 15. We validated DOX-induced downregulation of FUNDC1 and AAV9 injection-induced overexpression of FUNDC1 by immunoblotting (Figure 7A-B). Immunohistochemistry analysis confirmed FUNDC1 downregulation within cardiac tissue in the DOX group (Figure 7C). Apart from FUNDC1, we also validated the downregulation of multiple MERCs tethering proteins in myocardium of DOX-treated mice (Figure S7A-B). More importantly, FUNDC1 overexpression also ameliorated the disrupted MERCs structure within myocardium induced by DOX administration, which was confirmed by TEM (Figure 7D). To measure the autophagic flux *in vivo*, the mice were administrated with bafilomycin A1 before being sacrificed. We also found that the myocardial autophagic flux was blocked by DOX, while cardiac-specific overexpression of FUNDC1 corrected the dysregulated autophagic flux, as demonstrated by increased LC3 turnover (Figure 7E-G). Along with this, cardiac-specific overexpression of FUNDC1 increased LC3 puncta induced by bafilomycin A1 within myocardium after DOX treatment (Figure 7H). Besides, consistent with the in-vitro study, FUNDC1 overexpression enhanced myocardial ATG5-ATG12 and ATG16L1 level (Figure 7I). Overall, these data indicated that cardiac-specific overexpression of FUNDC1 restored the disrupted MERCs structure and blocked autophagic flux within myocardium in the DIC model.

### Cardiac-Specific overexpression of FUNDC1 ameliorated cardiac dysfunction and myocardial remodeling in DOX-treated mice

**Figure 8:**
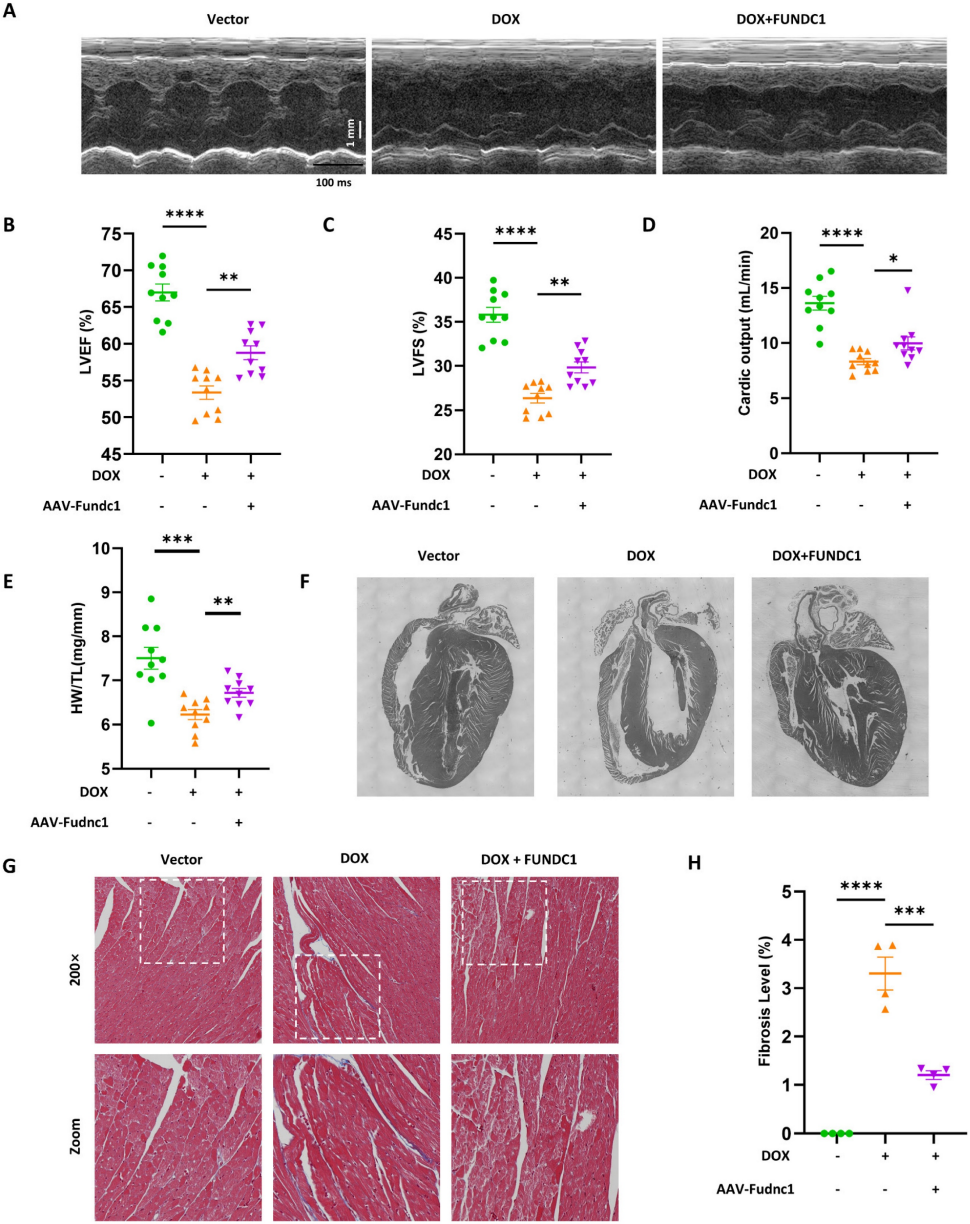
** Cardiac-specific overexpression of FUNDC1 ameliorated DOX-induced cardiac dysfunction and remodeling *in vivo*. (A)** Representative echocardiographic images of cardiac function; **(B-D)** Quantitative analysis of LVEF, LVFS and cardiac output (n = 10/group); **(E)** Heart weight (HW)-to-tibia length (TL) ratio was calculated; **(F)** An overview of mice heart in each group; **(G-H)** Representative images of Masson Trichrome staining and quantitative analysis of fibrosis level. (n = 4). * P < 0.05; ** P <0.01; *** P < 0.001; **** P < 0.0001.

To evaluate the cardioprotective effect of FUNDC1 in DIC *in vivo*, we determined the cardiac function using echocardiography. DOX treatment significantly impaired cardiac function, as demonstrated by reduced LVEF, LVFS and CO (Figure 8A-D). Cardiac-specific overexpression of FUNDC1 alleviated DOX-induced cardiac dysfunction. To assess the effect of FUNDC1 on cardiomyocytes death, Terminal Deoxynucleotidyl Transferase (TdT) dUTP Nick-End Labeling (TUNEL) assay was performed. To our surprise, DOX administration did not lead to significant TUNEL-positive cardiomyocytes in DIC mice (Figure S7C). This finding coincided with a previous study 25, which showed that DOX did not induce significant cardiomyocytes apoptosis, implying that other types of programed cell death may be involved. Although FUNDC1 overexpression didn't inhibit body weight loss induced by DOX treatment (Figure S7D), it significantly augmented heart weight-to-tibia length ratio (Figure 8E). This conformed to the improved myocardial structure due to FUNDC1 overexpression, including increased ventricle wall thickness and a concomitant reduction of the left ventricular internal diameter (Figure 8F). In addition, Masson's Trichrome Staining was applied to detect the cardiac fibrosis. DOX treatment led to an increase in cardiac fibrosis, which was alleviated by FUNDC1 overexpression (Figure 8G-H). Taken together, cardiac-specific overexpression of FUNDC1 exerted cardioprotective effect against DIC by ameliorating cardiac dysfunction and myocardial remodeling in DOX-treated mice.

## Discussion

Autophagy is a highly conserved lysosome-mediated intracellular degradation pathway and the dysregulation of autophagic flux has been proposed as mechanism of DOX-induced cardiotoxicity 3. However, due to the highly dynamics of autophagic flux, diversity of method for autophagic flux measurement as well as difference in concentration of DOX treatment, there is a discrepancy on the status of myocardial autophagic flux upon DOX treatment 18,26,27. Some studies identified the overactivation of autophagy was involved in the mechanism of DIC and inhibiting autophagy by Akt-mTOR pathway activation protected against DOX-induced cardiomyopathy 26,27. Similarly, another study also demonstrated that inhibition of autophagy by the transcription factor GATA4 antagonizes DIC 28. However, there are also some opposing views that DOX blocked myocardial autophagic flux either by inhibiting the lysosome acidification 17 or autophagy fusion process 18. Consistent with this, multiple studies demonstrated that the activation of autophagy was able to alleviate the DIC 29-32.

Due to these discrepancies, we meticulously investigated and comprehensively evaluated the dynamic changes of autophagic flux in cardiomyocytes subjected to DOX treatment with multiple approaches and methods. We firstly found that DOX treatment led to higher transcription of *MAP1LC3B* yet lower accumulation of LC3B II induced by CQ, suggesting that the elevated LC3BII induced by DOX was largely mediated by overall alterations in LC3 mRNA and protein levels, rather than by an effect on LC3 lipidation per se. This reminded us that autophagic flux may be blocked earlier before LC3 lipidation.

Moreover, the impaired PI3P activity and ATG5 puncta formation in DOX-treated cardiomyocytes under starvation conditions suggested the upstream of LC3 lipidation was also blocked by DOX. Since the LC3 lipidation contributed to the expansion of phagophore and autophagosome closure, it was expected that DOX treatment led to an inhibition on autophagosome formation. This is verified by reduced numbers and abnormal structures of autophagosomes upon autophagy induction in DOX treated cardiomyocytes. Overall, our study demonstrated that DOX treatment blocked autophagic flux by impairing autophagosome biogenesis.

Autophagy is initiated and autophagosomes are synthesized in a structure termed MERCs, where mitochondria and endoplasmic reticulum contact with each other 13. In the present study, we observed that multiple tethering proteins were downregulated at both transcriptional and translational levels. Therefore, the MERCs structure was expected to be disrupted by DOX treatment, as confirmed by confocal microscope and TEM. For the first time, we described the disruptive MERCs formation and compromised MERCs function in DOX-treated cardiomyocytes. Since MERCs were reported as the sites where autophagosomes form, a variety of studies have verified the regulatory role of MERCs tethering proteins in autophagy 33. PACS2, one of tethering protein of MERCs was reported to promote the formation of mitophagosome in vascular smooth muscle cell and renal tubule cell 34,35. Sigma-1 receptor is another tethering protein of MERCs. Activation of sigma-1 enhanced autophagy, while its ablation blocked autophagic flux 36,37. Besides, we also previously demonstrated that MFN2 acts as an important modulator of MERCs dynamics and plays an important role in autophagy induced by energy stresses 38. While MERCs formation is generally believed to be the facilitator of autophagy, some other studies described that disruption of MERCs by heavy metal, including cadmium and copper, unexpectedly activated autophagy 39-41. Despite these discrepancies, all of these studies verified the essential role of MERCs in autophagy. Our present study demonstrated that FUNDC1, one of MERCs tethering proteins, functioned as a key component regulating MERCs structures and function in DIC. Notably, restoration of MERCs by FUNDC1 also corrected the blocked autophagic flux induced by DOX. Since autophagosomes are synthesized on MERCs structure, it is presumptive that restoration of MERCs by FUNDC1 overexpression is able to increase the number of autophagosomes. But to our surprise, the overexpression of FUNDC1 also increases the autolysosome-to-autophagosome ratio, indicating enhanced autophagosome turnover. These data suggested that FUNDC1 may also potentially promote the clearance of autophagosome to facilitate autophagic flux either by accelerating the fusion between autophagosome and lysosome or ameliorating lysosome dysfunction in DOX-treated cardiomyocytes. It was worth noting that our study identified that FUDNC1 augmented ATG5-ATG12 complex and enhanced ATG5 puncta formation, which was not only vital for autophagosome formation, but also defined to be necessary for the activation and repair of lysosome 42,43. This may potentially explain the dual effects of FUNDC1 on both early and late stage of autophagic flux. The proposed hypothesis remains to be an important direction for our future study.

Another interesting point deserved discussion is that despite FUNDC1 acting as a mitophagy receptor as well, we did not observe any detectable effect of FUNDC1 overexpression on mitophagic flux in DOX-treated cardiomyocytes. It is believed that different subtypes of mitophagy may dominate in a specific context responsible for mitochondrial quality control. So far, the Parkin-meditated mitophagy and BNIP3-meditated mitophagy are the main kinds of mitophagy identified in DIC 2, while FUNDC1 plays an important role in hypoxia-induced mitophagy 7 and exerts a role in pathogenesis of cardiac ischemia reperfusion injury 44. Besides, induction of FUNDC1-meditated mitophagy requires modulation of phosphorylation and dephosphorylation of FUNDC1 by Src/CK2/ULK1 and PGAM5 45. In this study, we expressed unmodified FUNDC1 and the expression of kinase and phosphatase in charge of post-translational modification of FUNDC1 remained unknown upon DOX treatment, which may potentially provide another explanation that FUNDC1 overexpression did not alter the mitophagic flux in DOX-treated cardiomyocytes. The specific underlying mechanism deserves further investigation. Nevertheless, we demonstrated that FUDNC1 restored the MERCs structure and corrected the dysregulated autophagic flux in DIC. To the best of our knowledge, this is the first study to reveal that FUNDC1 can enhance macroautophagy via restoring MERCs structure in the DIC model, apart from its regulatory role as an mitophagy receptor.

Autophagy is a major sensor of redox signaling, implicated in stress responses, elimination of damaged organelles, and programmed cell death 46. In the present study, we demonstrated that FUNDC1 ameliorated oxidative stress induced by DOX. More importantly, we also found that FUNDC1 inhibited DOX-induced cardiomyocytes death and alleviated the reduction in cell viability in an autophagy-dependent manner. The modulation of FUNDC1 on autophagy was also validated *in vivo*. Cardiac-specific overexpression of FUNDC1 ameliorated cardiac dysfunction and remodeling in DOX-treated mice. These results unveiled the protective effect of FUNDC1 against DIC. Consistent with our study, the cardioprotective effect of FUNDC1 against DIC has been also revealed by another group, in which FUNDC1 inhibited PANoptosis by stabilizing mtDNA via interaction with TUFM during the implementation of our study 47. Nevertheless, the FUNDC1-medicated autophagy and MERCs involvement in DIC are firstly identified in our present study.

## Conclusion

In summary, we herein demonstrated that DOX significantly inhibited autophagosome biogenesis, which was associated with disrupted MERCs structure and compromised MERCs function. We also revealed a novel role of FUNDC1, enhancing autophagy as a MERCs tethering protein, which exerted cardioprotective effect against DIC. This finding is of paramount significance in deciphering the molecular mechanism involved in DIC and potentially shed some light on the prevention and treatment of DIC in clinical practice.

## Supplementary Material

Supplementary figure legends and tables.

Supplementary figures.

## Abbreviations

DOX: Doxorubicin
MERCs: Mitochondrial-Endoplasmic Reticulum Contacts
DIC: DOX-induced cardiotoxicity
ROS: Reactive Oxygen Species
MDA: Malondialdehyde
LVEF: Left Ventricular Ejection Fraction
LVFS: Left Ventricular Fraction Shortening
CO: Cardiac Output
