# Supplementary material for: FUNDC1 alleviates doxorubicin-induced cardiotoxicity by restoring mitochondrial-endoplasmic reticulum contacts and blocked autophagic flux
Source: Theranostics. 2024 Jun 17;14(9):3719–38. doi: 10.7150/thno.92771 (PMC11209712; doi:10.7150/thno.92771)
Supplement: Supplementary file 1 — Supplementary figure legends and tables. [file thnov14p3719s1.pdf]

Supplementary Figure Legend

Fig S1 (A-C) AC16 were treated with indicated concentration of DOX for 24 h, MFN1, MFN2, and IP3R1 were detected with immunoblotting and quantified, (n = 4); (D) AC16 were treated with 2 μM DOX for 0, 6, 12, 24,48 h, MFN1, MFN2, FUNDC1 and IP3R1 were detected with immunoblotting; (E-F) Representative images of cytosolic calcium stained by Fluo-4. Scale bar: 20 μm, mean fluorescence intensity were quantified in each group, (n = 5); (G) AC16 were treated with 2 μM DOX for 24 h, cytosolic calcium was labeled by Fluo-4, cells were stimulated with 0.1 mM ATP, △F/F0 were calculated to indicated calcium flux. (n = 6 cells/group); ns: no significance; * P < 0.05; ** P <0.01; *** P < 0.001, DOX: doxorubicin.

Fig S2 (A-B) AC16 were transfected with ER-DsRed for 24 h followed by 2 μM DOX treatment for 24 h, immunostaining of TOM20 indicated mitochondria, images were acquired with confocal microscope. Scale bar:5 μm, Pearson's coefficients indicating colocalization of mitochondria and endoplasmic reticulum were quantified, (n = 19-30 cells/group); (C) AC16 were transfected with Vector, FUNDC1 and FUNDC1△, immunostaining of TOM20 indicated mitochondria, images were acquired with confocal microscope, FLAG indicated FUNDC1 or FUNDC1 △. Scale bar:5 μm; (D-E) Representative images of cytosolic calcium stained by Fluo-4. Scale bar: 20 μm, mean fluorescence intensity were quantified in each group, (n = 5); (F) AC16 were treated with 2 μM DOX for 24 h, cytosolic calcium was labeled by Fluo-4, cells were stimulated with 0.1 mM ATP, △F/F0 were calculated to indicated calcium flux. (n = 6 cells/group); (G) mito-R-GECO1 stable cell line were infected with FUNDC1 lentivirus, cells were lysed and subjected to immunoblotting. ns: no significance; **** P < 0.0001, DOX: doxorubicin.

Fig S3 (A) AC16 were transfected with Vector, FUNDC1 and FUNDC1 △ plasmid for 24 h followed by 2 μM DOX treatment for 24 h and CQ treatment for 2 h, LC3B were detected with immunoblotting; (B) Quantitative analysis of LC3B II (n = 4); (C)

Vector or FUNDC1 stable cell lines were treated with 2 μM DOX for 24 h, mRNA level of MAP1LC3B were detected with qPCR, (n = 6); (D) AC16 were transfected with EGFP-ATG5 for 24 h followed by 2 μM DOX treatment for 24 h and starvation for 2 h. Images were acquired with confocal microscope. Scale bar:5 μm; ATG5 puncta in each group were quantified (n = 11-27 cells/group); (E) AC16 were transfected with EGFP-FYVE for 24 h followed by 2 μM DOX treatment for 24 h and starvation for 2 h. Images were acquired with confocal microscope. Scale bar:5 μm; (F) Vector or FUNDC1 stable cell lines were transfected with EGFP-FYVE for 24 h followed by 2 μM DOX treatment for 24 h and starvation for 2 h. Images were acquired with confocal microscope. Scale bar:5 μm; ns: No significance, *** P < 0.001; **** P < 0.0001; DOX: doxorubicin.

Fig S4 (A) AC16 were infected with lentivirus targeting FUNDC1, cells were lysed and immunoblotting for FUNDC1; (B) FUNDC1 overexpression (OE) and knock-down (KD) stable cell lines were treated with 2 μM DOX treatment for 24 h, ATG5-ATG12 and ATG16L1 were detected with immunoblotting; (C) Quantitative analysis of ATG5-ATG12 (n = 3); (D) Quantitative analysis of ATG16L1 (n = 3); *** P < 0.001; **** P < 0.0001; DOX: doxorubicin.

Fig S5 (A) AC16 were transfected with EGFP-LC3 for 24 h followed by 2 μM DOX treatment for 24 h. Mitochondria were stained by Mito-tracker. Images were acquired with confocal microscope. Scale bar:10 μm, The LC3 puncta colocalized with mitochondria in each cell were quantified (n = 15-20 cells/group); (B) AC16 were transfected with mCherry-EGFP-FIS1 for 24 h followed by 2 μM DOX treatment for 24 h. Mitophagy events indicated by red-shifted dots were quantified in each cell (n = 10-20 cells/group); (C) AC16 were treated with 2 μM DOX for 24 h, FLAG, HSP60, COX IV, and TOM20 were detected with immunoblotting followed by quantitative analysis in (D) (n = 3); ns: No significance, * P < 0.05; ** P <0.01; **** P < 0.0001; DOX: doxorubicin.

Fig S6 (A) Vector or FUNDC1 knock-down (KD) stable cell lines were treated with 2 μM DOX for 24 h followed by ROS measurement with DCFH-HA staining; (B) Relative ROS level was quantified in each group, (n = 6); (C) AC16 were infected with lentivirus targeting ATG5, cells were lysed and immunoblotting for ATG5; (D) Vector or FUNDC1 knock-down (KD) stable cell lines were treated with 2 μM DOX for 24 h, relative MDA level in each group were quantified, (n = 6); (E) Vector or FUNDC1 knock-down (KD) stable cell lines were treated with 2 μM DOX for 24 h, SOD activities were determined in each group. (n = 6); (F) Vector or FUNDC1 knock-down (KD) stable cell lines were treated with 2 μM DOX for 24 h, cell death was indicated by SYTOX Green staining and quantified in (G), (n = 6); (H) Cell viability was determined by CCK-8 assay (n = 6); ns: no significance; * $P < 0.05$; ** $P < 0.01$; **** $P < 0.0001$; DOX: doxorubicin.

Fig S7 (A) qPCR analysis of mRNA of MERCs tethering proteins in heart tissue; (B) Immunostaining of MFN1, MFN2, and IP3R1 in heart tissue in each group; (C) TUNEL staining of paraffin-embedded heart tissue sections; (D) Record of bodyweight since AAV-9 injection. ** $P < 0.01$; DOX: doxorubicin.

Supplemental Table 1 The primers used in this study

**Gene** (Homo sapiens)	Forwards	Reverse
FUNDC1	CCCCCTCCCCAAGACTATGA	AGAAAGCCACCACCTACTGC
MFN1	ACGCCTTAGTGCTTCAGACC	GTACAAGGGAAGACCAGCCC
MFN2	CACAAGGTGAGTGAGCGTCT	CGTTGAGCACCTCCTTAGCA
ITPR1	GAGATTCGCAGCAAGAGTG	CTCGCAAAGAGGTTCAGC
ITPR2	CAACGCCAAGGTTTAAGG	TGGAAGGATTGGTTCAGG
VDAC1	CCTTTGAGATGCCAGGTTT	TTATCCGCTTCCACTTGC
GRP75	TGAAACCATCTCGCACAC	TCAGTTGAAGGACCCCAT
MAP1LC3B	GTCAGCGTCTCCACACCAAT	TTCATCCCGAACGTCTCCTG
GAPDH	CAAGAAGGTGGTGAAGCAGG	CCACCCTGTTGCTGTAGCC

Supplemental Table 2 The primers used in this study

**Gene** (Mus musculus)	Forwards	Reverse
Fundc1	TGTAATGGGTGGCGTGACTG	CCACGAAGCCGCTGGATATC
Mfn1	GATAAAGTCCTCCCCAGCGG	GGTGACATCTGTACCTGGGC
Mfn2	TCGGAGCCTGAGTACATGGA	GCACTCCTCAAACTGCCTCT
Itpr1	GGGGAGGATGAGGAAGAGGT	AGGTCGTAGGGGAGGTTCTC
Itpr2	CTGCTGAAAAACATGGGGGC	CATGAAGATGTGCCGCATGG
Vdac1	CGTGGACTGAAGCTCACCTT	AAGTTGCTCTGGGTCACTCG
Grp75	TGTCACTCCCCTCTCTCTGG	TTCAATCTGGGGCACTCCAC
Gapdh	CAAGAAGGTGGTGAAGCAGG	CCACCCTGTTGCTGTAGCC
