# Supplementary figures and images for: FUNDC1 alleviates doxorubicin-induced cardiotoxicity by restoring mitochondrial-endoplasmic reticulum contacts and blocked autophagic flux
Source: Theranostics. 2024 Jun 17;14(9):3719–38. doi: 10.7150/thno.92771 (PMC11209712; doi:10.7150/thno.92771)

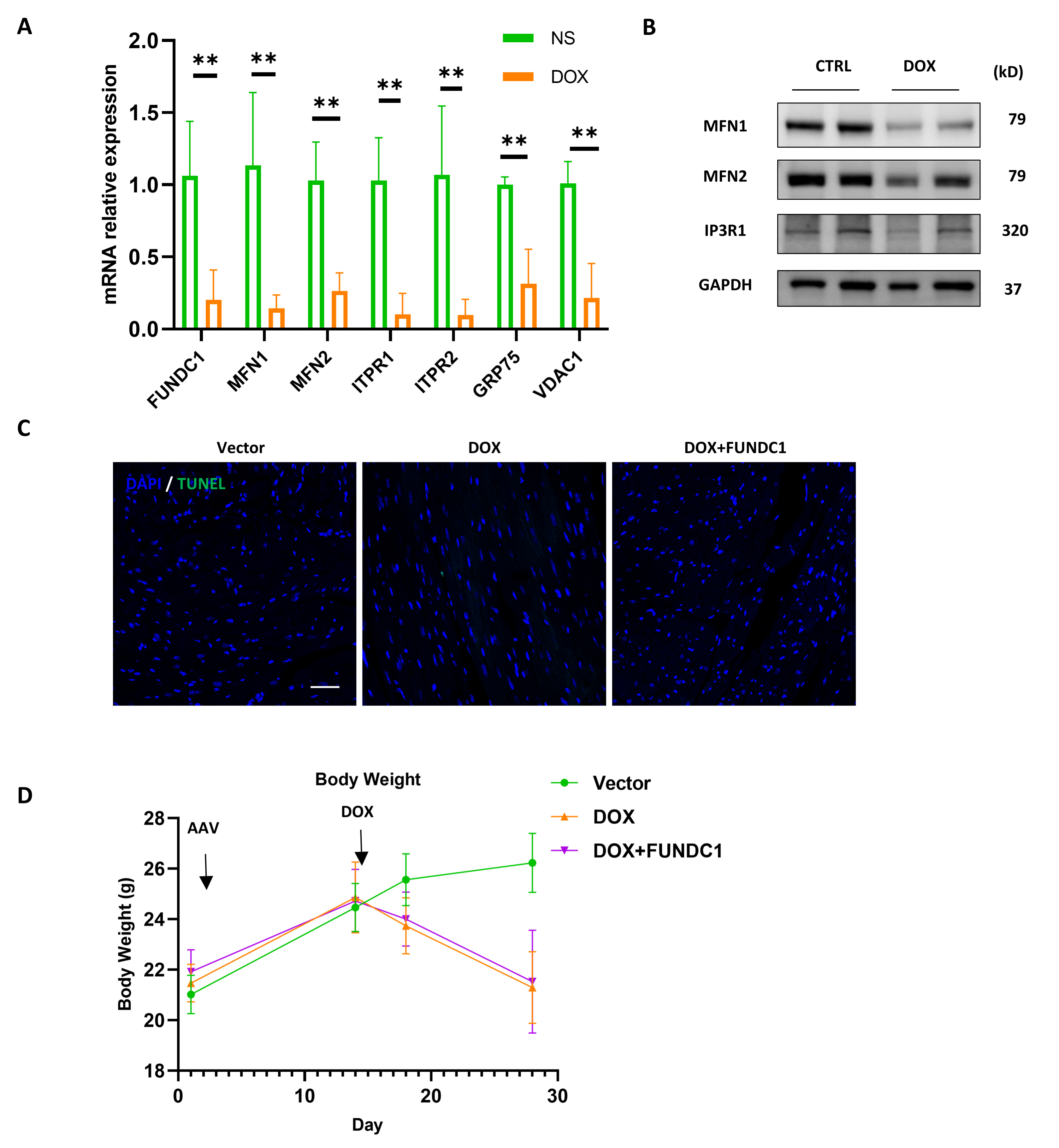

Supplement: Supplementary file 2 — Supplementary figures. [file thnov14p3719s2.zip › Supplemental Figures/Figure S7.tif]

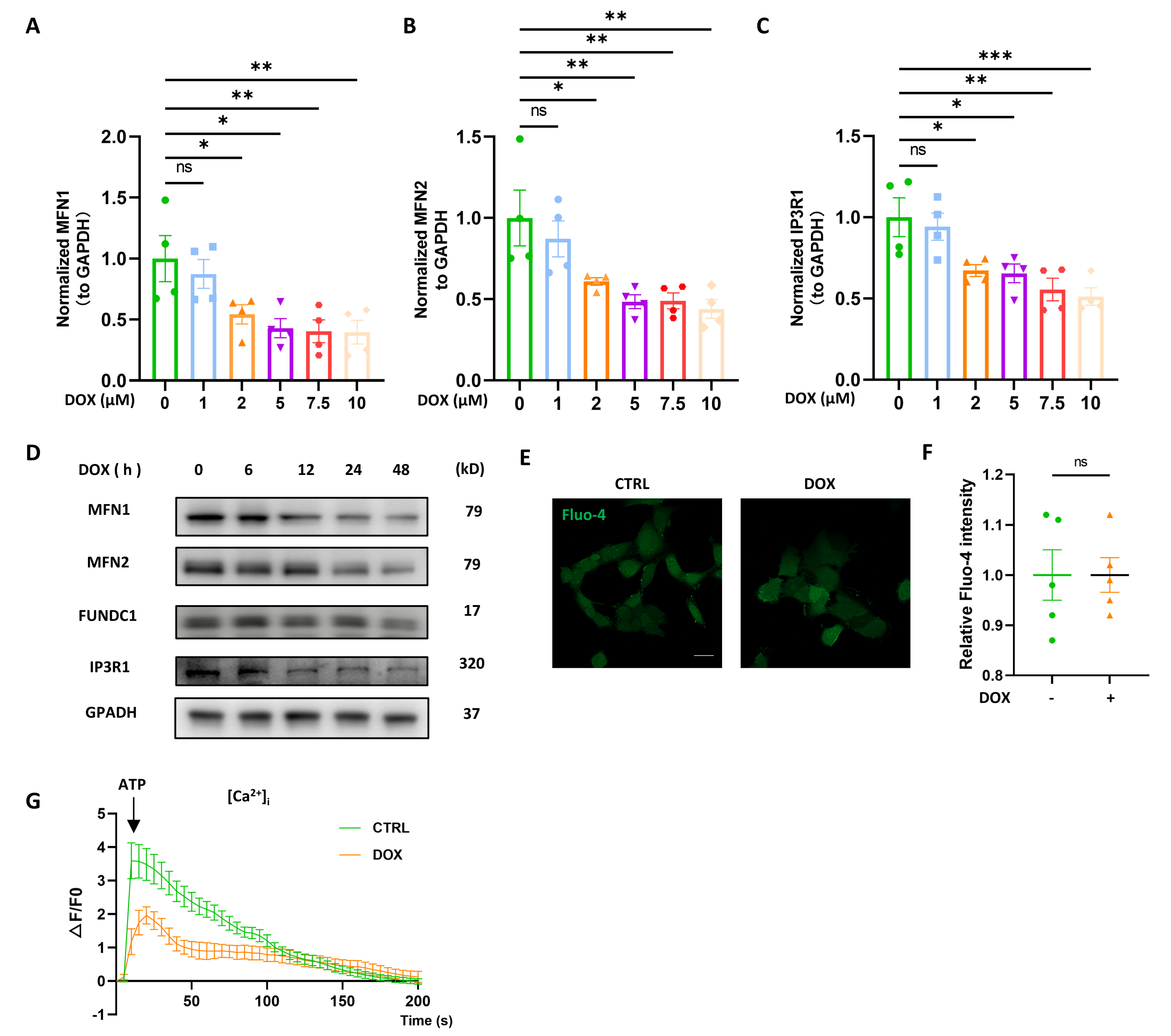

Supplement: Supplementary file 2 — Supplementary figures. [file thnov14p3719s2.zip › Supplemental Figures/Figure S1.tif]

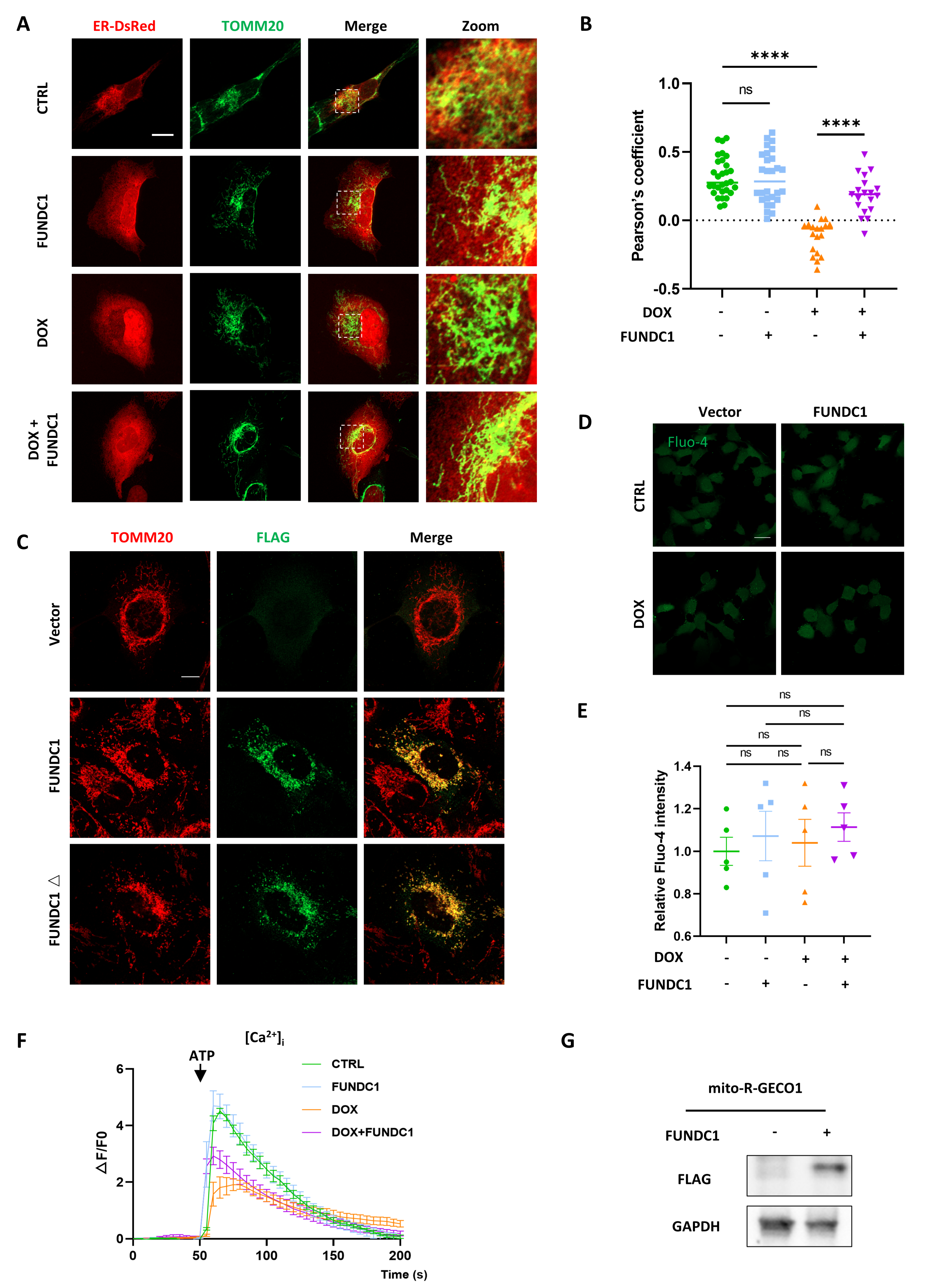

Supplement: Supplementary file 2 — Supplementary figures. [file thnov14p3719s2.zip › Supplemental Figures/Figure S2.tif]

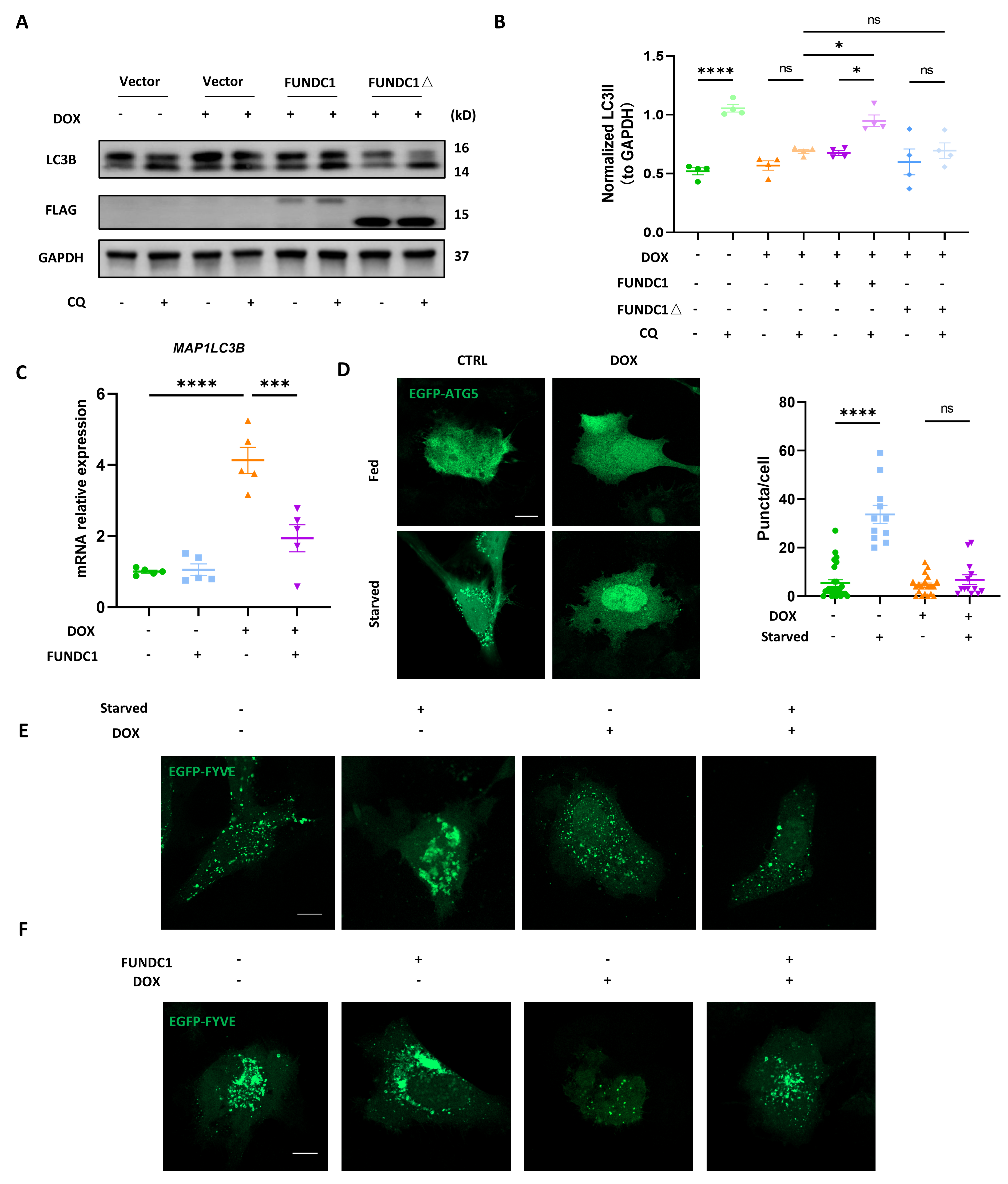

Supplement: Supplementary file 2 — Supplementary figures. [file thnov14p3719s2.zip › Supplemental Figures/Figure S3.tif]

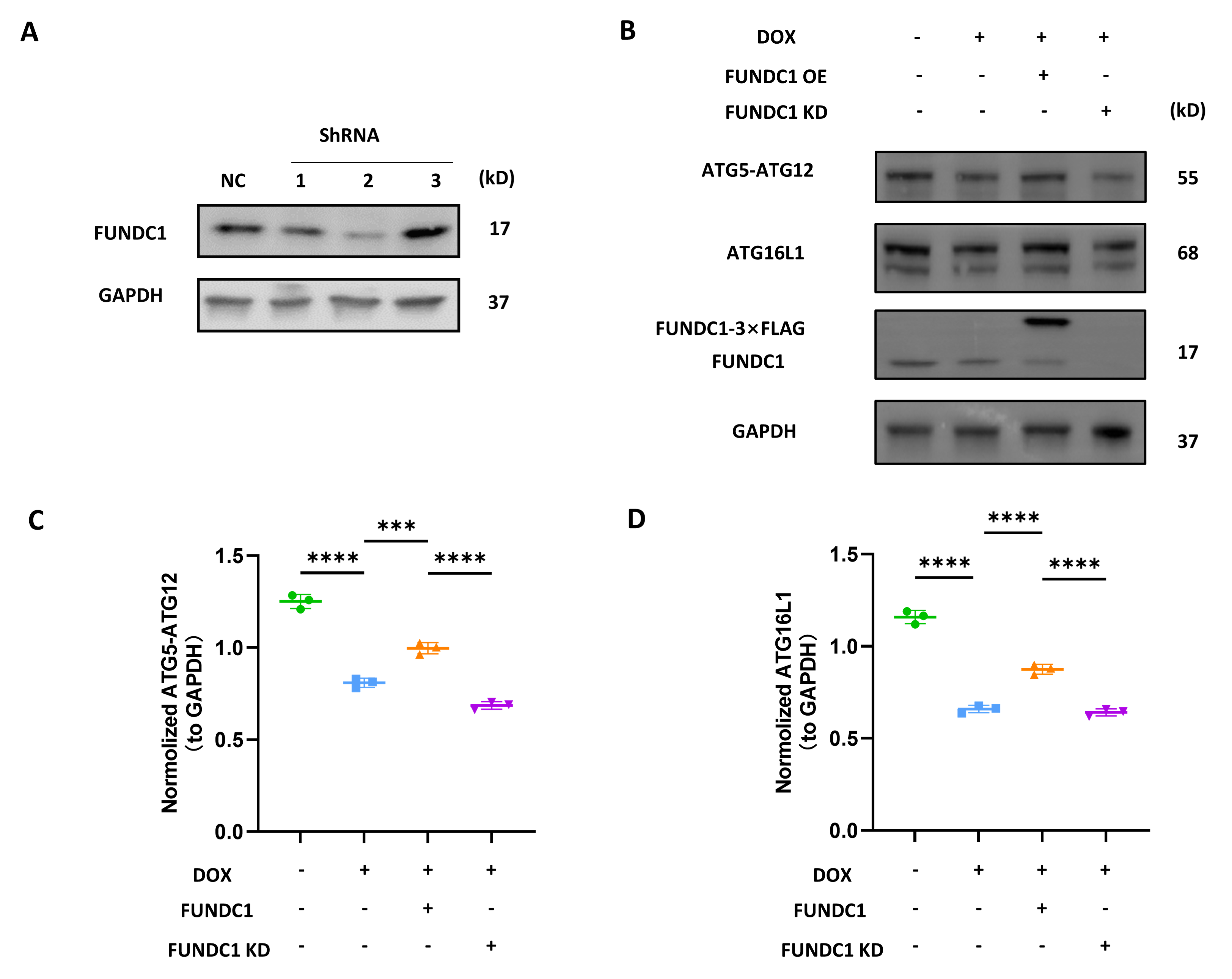

Supplement: Supplementary file 2 — Supplementary figures. [file thnov14p3719s2.zip › Supplemental Figures/Figure S4.tif]

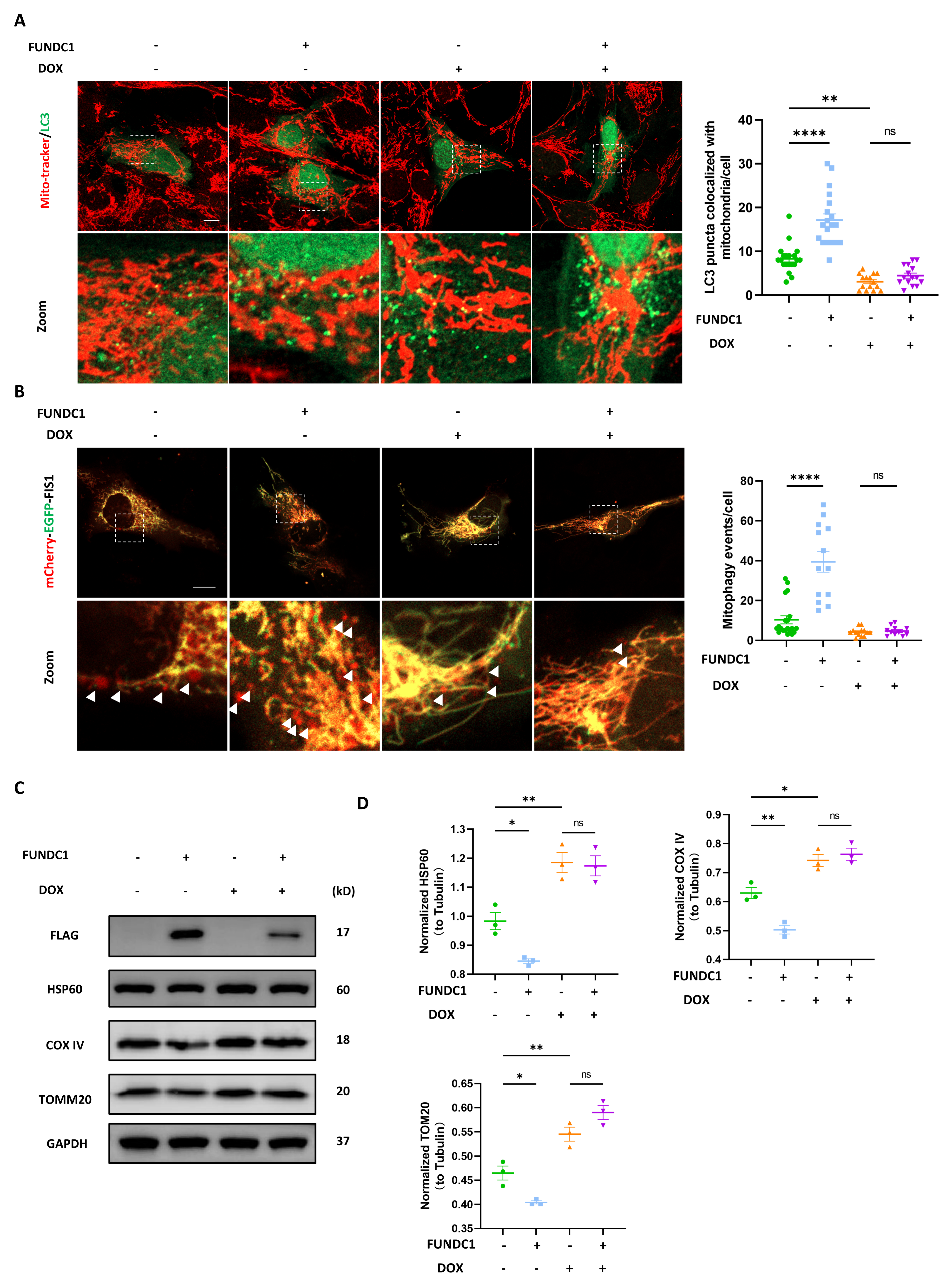

Supplement: Supplementary file 2 — Supplementary figures. [file thnov14p3719s2.zip › Supplemental Figures/Figure S5.tif]

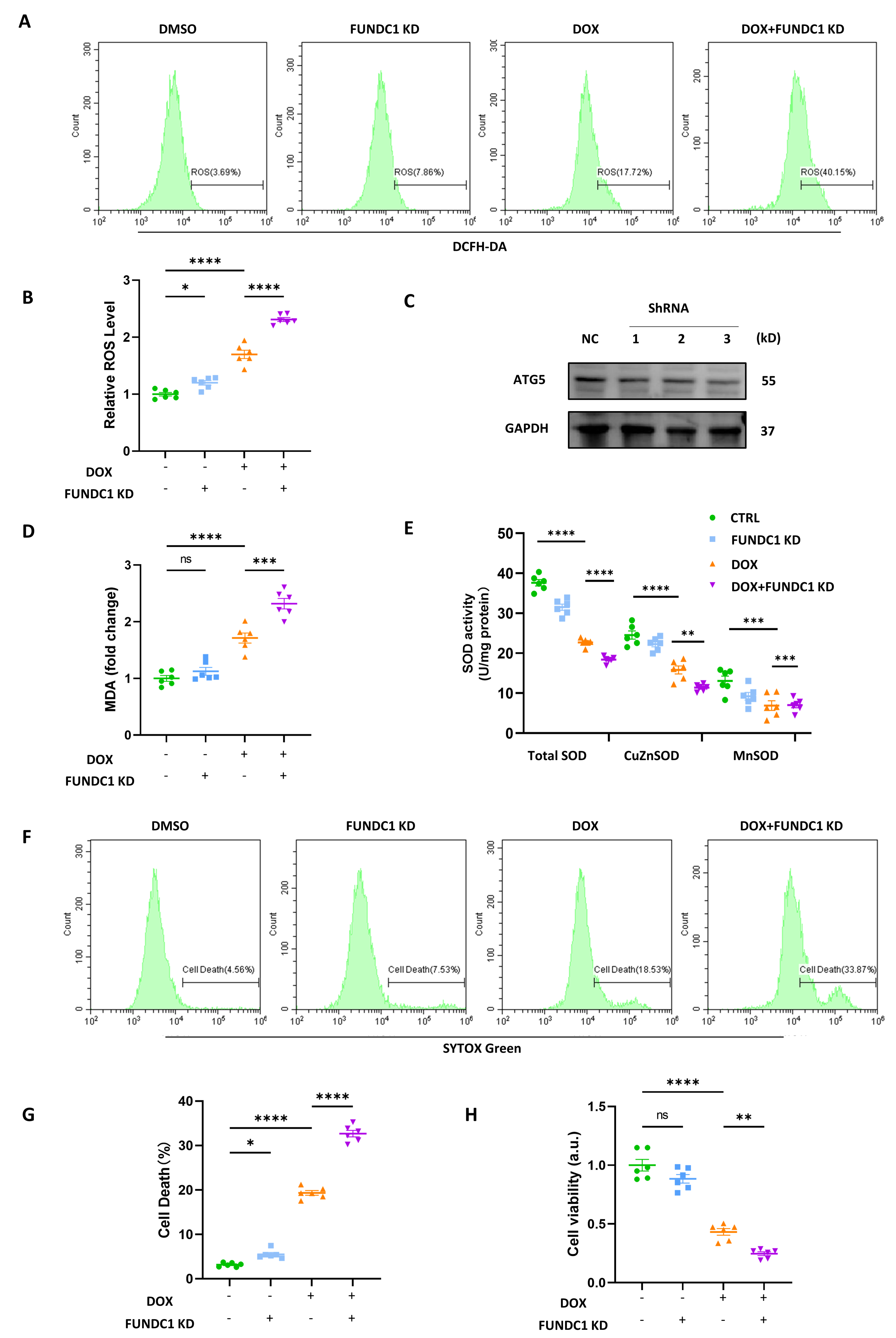

Supplement: Supplementary file 2 — Supplementary figures. [file thnov14p3719s2.zip › Supplemental Figures/Figure S6.tif]
